# Supplementary material for: Glyoxal as an alternative fixative to formaldehyde in immunostaining and super‐resolution microscopy
Source: EMBO J. 2017 Nov 16;37(1):139–59. doi: 10.15252/embj.201695709 (PMC5753035; doi:10.15252/embj.201695709)
Supplement: Supplementary file 1 — Appendix [file EMBJ-37-139-s001.pdf]

Appendix for
Glyoxal as an alternative fixative to formaldehyde in immunostaining and super-resolution microscopy

Katharina N. Richter[1,2†], Natalia H. Revelo[1,†,‡], Katharina J. Seitz[1,3], Martin S. Helm[1,3], Deblina Sarkar[4], Rebecca S. Saleeb[5], Elisa D'Este[6], Jessica Eberle[7], Eva Wagner[8,9], Christian Vogl[10,11], Diana F. Lazaro[12,13], Frank Richter[3,14], Javier Coy-Vergara[15], Giovanna Coceano[16], Edward S Boyden[17], Rory R. Duncan[5], Stefan W. Hell[6], Marcel A Lauterbach[7], Stephan E. Lehnart[8,9], Tobias Moser[10,11], Tiago Outeiro[12,13], Peter Rehling[14,18], Blanche Schwappach[15], Ilaria Testa[16], Silvio O. Rizzoli[1,2*]

1 Department of Neuro- and Sensory Physiology, University of Göttingen Medical Center, Göttingen, Germany

2 Cluster of Excellence Nanoscale Microscopy and Molecular Physiology of the Brain, Göttingen, Germany

3 International Max Planck Research School Molecular Biology, Göttingen, Germany

4 MIT Media Lab

5 Edinburgh Super-Resolution Imaging Consortium, Institute of Biological Chemistry, Biophysics, and Bioengineering, Heriot-Watt University, Edinburgh, UK

6 Department of NanoBiophotonics, Max-Planck-Institute for Biophysical Chemistry, Göttingen, Germany

7 Department of Neural Systems, Max-Planck-Institute for Brain Research, Frankfurt am Main, Germany

8 Heart Research Center Göttingen, Department of Cardiology & Pulmonology, University Medical Center Göttingen, Göttingen, Germany

9 German Center for Cardiovascular Research (DZHK) Site Göttingen

10 Institute for Auditory Neuroscience and InnerEarLab, University Medical Center Göttingen, Göttingen, Germany

11 Max-Planck-Institute for Experimental Medicine, Auditory Neuroscience Group, Göttingen, Germany

12 Department of Experimental Neurodegeneration, Center for Nanoscale Microscopy and Molecular Physiology of the Brain, University Medical Center Göttingen, Göttingen, Germany

13 Max-Planck-Institute for Experimental Medicine, Göttingen, Germany

14 Department of Cellular Biochemistry, University Medical Center Göttingen, Göttingen, Germany

15 Department of Molecular Biology, University Medical Center Göttingen, Göttingen, Germany

16 Department of Applied Physics and Science for Life Laboratory, KTH Royal Institute of Technology, Stockholm, Sweden

17 Departments of Brain and Cognitive Science and Biological Engineering, MIT Media Lab and McGovern Institute, Cambridge, MA, USA

18 Max-Planck-Institute for Biophysical Chemistry, Göttingen, Germany

19 Max Planck Research Unit for Neurogenetics, Frankfurt am Main, Germany

*Correspondence should be addressed to Silvio O. Rizzoli (srizzol@gwdg.de)

†these authors contributed equally

‡Current address: Department of Tumor Immunology, Radboud Institute for Molecular Life Sciences, Radboud University Medical Center, Nijmegen, the Netherlands

Table of contents

Appendix Figure S1 Addition of ethanol increases the speed of membrane penetration, but neither ethanol nor low pH improves immunostainings . 3

Appendix Figure S2 Fixation of Alexa Fluor 594-coupled transferrin, applied during fixation. 4

Appendix Figure S3 Maintenance of the acidic lumen of lysosomes after fixation with PFA or glyoxal for different durations. 5

Appendix Figure S4 Transferrin and cholera toxin uptake during fixation. 6

Appendix Figure S5 Preservation of mitochondria after 60 min of fixation. 7

Appendix Figure S6 Preservation of cellular structures by PFA and glyoxal fixation. 8

Appendix Figure S7 Comparison of glyoxal fixation with various alternatives of PFA fixation. 9

Appendix Figure S8 Fixation of lipids by PFA and glyoxal. 10

Appendix Figure S9 Preservation of enzymatic activity after PFA and glyoxal fixation. 11

Appendix Figure S10 Immunostaining efficiency after PFA and glyoxal fixation. 12

Appendix Figure S11 Preservation and antibody recognition of cellular targets fixed by PFA and glyoxal. 13

Appendix Figure S12 Super-resolution imaging of 20 immunostained proteins in cultured neurons after fixation with PFA or glyoxal. 14

Appendix Figure S13 Ten pairs of average objects (structures) from neurons immunostained after PFA or glyoxal fixation. 16

Appendix Figure S14 Ten additional pairs of averaged objects from neurons immunostained after PFA or glyoxal fixation (continuation of Appendix Figure S13). 18

Appendix Figure S15 Electron microscopy of PFA- or glyoxal-fixed hippocampal neurons. 19

Appendix Figure S16 Glyoxal fixation in tissue from *Drosophila* larvae. 20

Appendix Figure S17 Glyoxal fixation in mammalian tissue: mouse inner hair cells. 21

Appendix Figure S18 Glyoxal fixation in mammalian tissue: mouse neuromuscular junctions. 22

Appendix Table 1: Qualitative analysis of different fixation conditions. 23

Methods of collaborating laboratories 26

References 33

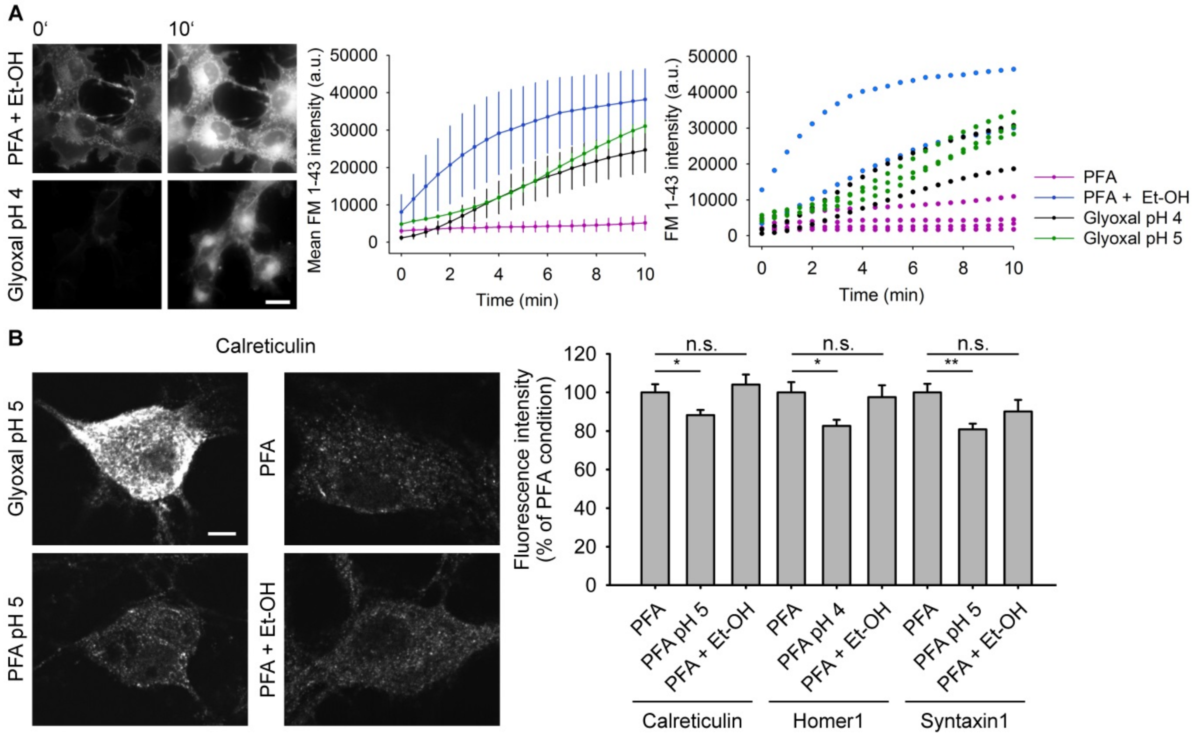

Appendix Figure S1 Addition of ethanol increases the speed of membrane penetration, but neither ethanol nor low pH improves immunostainings .

A The images show the entry of FM 1-43 into fibroblasts during the first 10 min of fixation. In addition to fixation with PFA and glyoxal pH 5 (as shown in Fig. 1b), we tested fixation with glyoxal pH 4, and with PFA + ethanol. In the graphs either individual data points or mean values with SEM are plotted. N = 2 independent experiments for glyoxal pH 4 and for PFA + ethanol. Scale bar = 20 µm.

B For testing the influence of EtOH and low pH on fixation with PFA and immunostaining, primary hippocampal neurons were fixed either with conventional PFA buffers (no ethanol, pH 7), with PFA containing the same amount of EtOH as our glyoxal-based fixatives, or with PFA at low pH. Immunostainings for calreticulin, homer 1 and syntaxin 1 show that neither the addition of EtOH, nor the low pH increase the quality of immunostainings. The quantification of fluorescence intensity even shows a reduced signal for samples fixed with PFA at low pH. N = 9 – 15 cell regions per condition (calreticulin, syntaxin 1), focusing on large areas that contained the widely diffused signal for these two proteins. For homer 1 we analyzed smaller regions containing synapses; 68 – 80 cell regions per condition. Scale bar = 5 µm. * $p < 0.05$, ** $p < 0.01$.

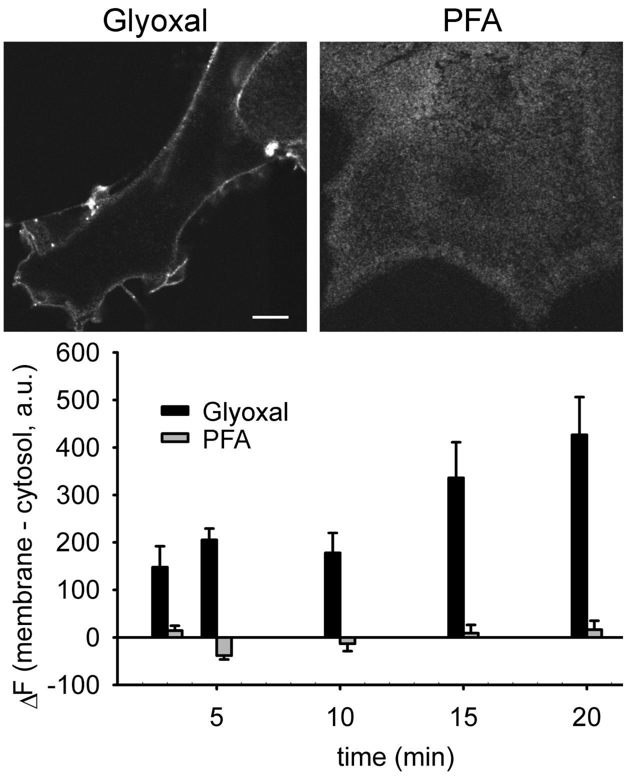

Appendix Figure S2 Fixation of Alexa Fluor 594-coupled transferrin, applied during fixation.

COS-7 cells were fixed at 37 °C for 3, 5, 10, 15 and 20 min, before transferrin-Alexa Fluor 594 was added, and the cells were incubated in the fixative and transferrin for another 20 min. The fluorescence intensity was measured in the cytosol of the cells and on the plasma membrane. The graph shows the fluorescence intensity at the membrane, from which the fluorescence intensity in the cytosol was subtracted. This analysis reveals that transferrin is fixed on the plasma membrane by glyoxal, whereas PFA-fixed cells show transferrin both on the plasma membrane and in the cytosol. N = 8-24 cellular ROIs per condition. Scale bar = 5 µm.

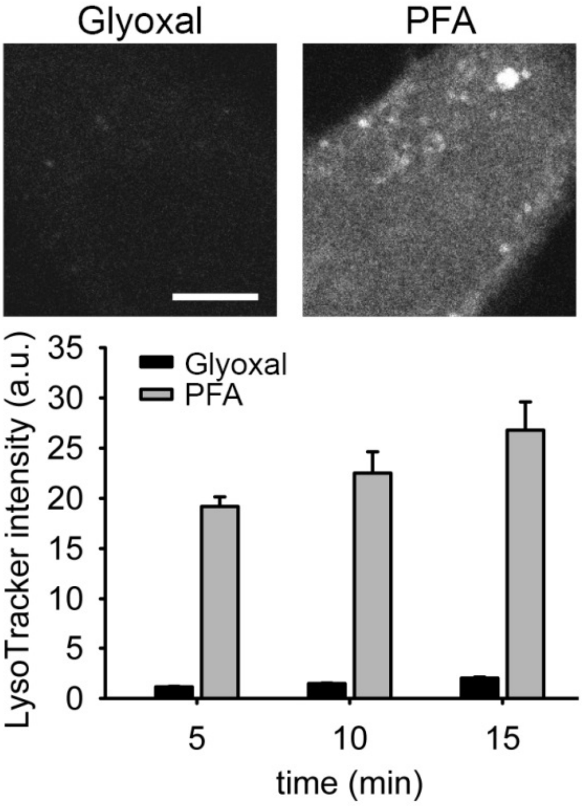

Appendix Figure S3 Maintenance of the acidic lumen of lysosomes after fixation with PFA or glyoxal for different durations.

HeLa cells were fixed at 37 °C for 5, 10 and 15 min before LysoTracker was added, and the cells were incubated in the fixative and LysoTracker for another 20 min. The measured LysoTracker intensity reveals the maintenance of the acidic lumen of lysosomes in PFA-fixed cells, whereas in glyoxal-fixed cells the acidic compartments are not detected. N = 9-21 cells per condition. Scale bar = 5 µm.

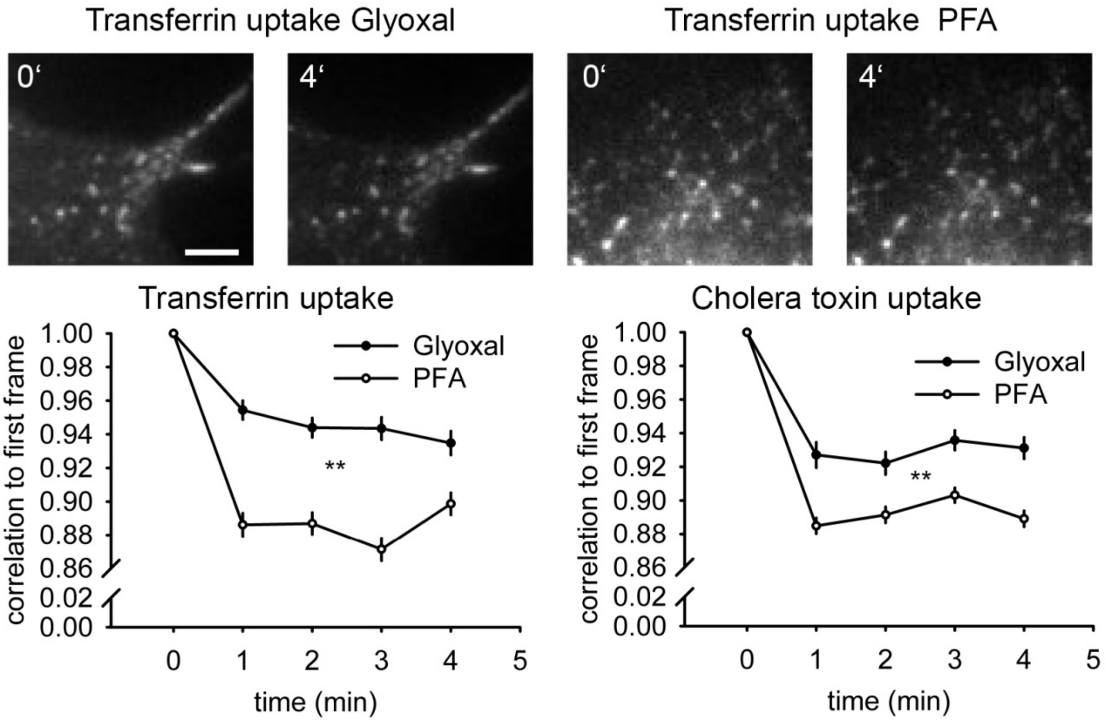

Appendix Figure S4 Transferrin and cholera toxin uptake during fixation.

COS-7 and HeLa cells were incubated with transferrin-Alexa Fluor594 or cholera toxin subunit B-Alexa Fluor555 for 10 min at 37 °C, and were imaged during the first few min after the addition of the fixative, during which time it is likely that active cellular movements still take place. Representative images are shown from the transferrin uptake experiment. The graphs show the correlation to the first frame of each image taken throughout the first minutes of fixation. The high correlation coefficients show that both fixatives allow little endosome movement, though glyoxal seems to act slightly faster than PFA. N = 49-325 cellular regions from 3 - 4 independent experiments. Scale bar = 5 µm. **p < 0.01

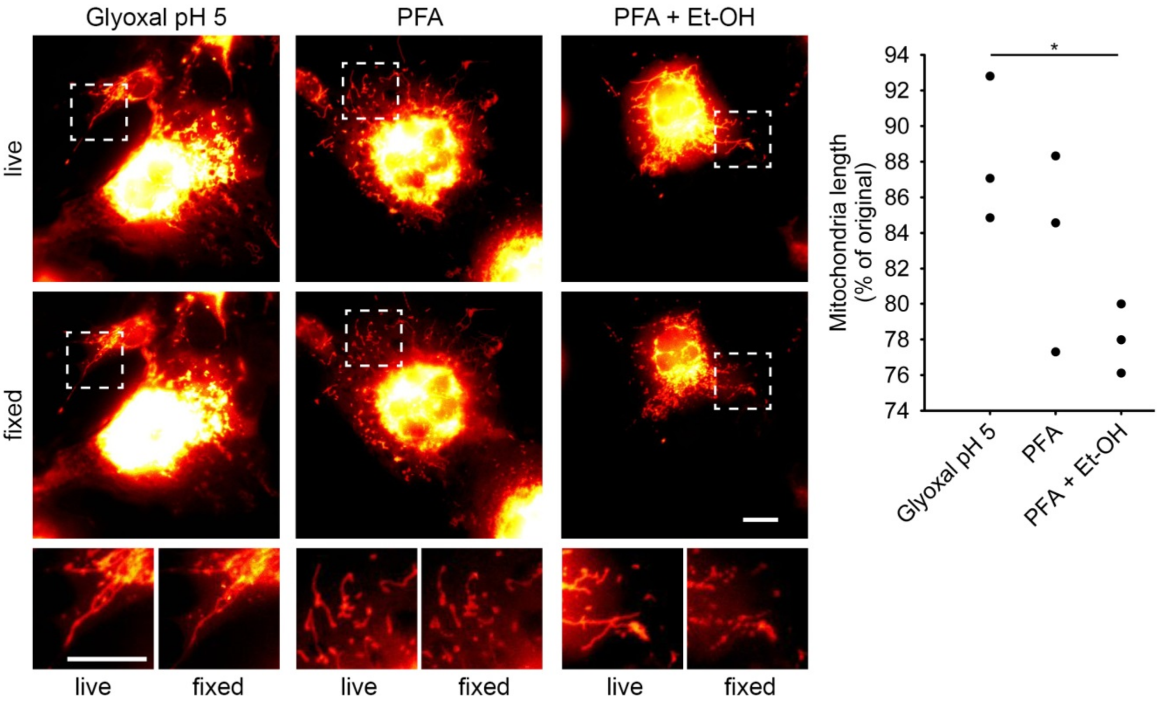

Appendix Figure S5 Preservation of mitochondria after 60 min of fixation.

Fibroblasts were transfected with the mitochondrial marker TOMM70, linked to GFP, and were imaged before and after fixation. The graph indicates the decrease in mitochondria size, caused by fragmentation during fixation (visible in the zoom-ins at the bottom of the panels). The mitochondria preservation was not significantly different between PFA and glyoxal, but was significantly different between glyoxal and PFA with added ethanol. This suggests that the ethanol addition to PFA does not increase its fixation accuracy. N = 3 experiments. Scale bar = 20 µm. *$p < 0.05$.

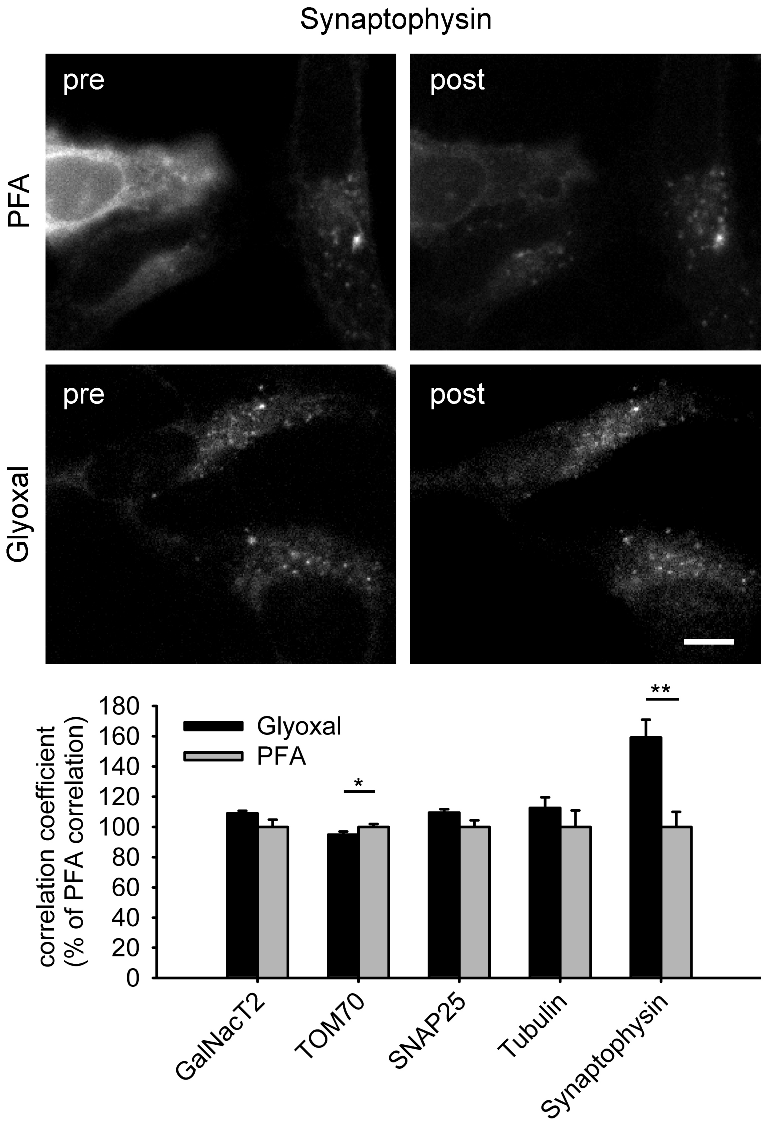

Appendix Figure S6 Preservation of cellular structures by PFA and glyoxal fixation.

Various GFP-tagged proteins were expressed in COS-7 and HeLa cells. The preservation of structures/organelles was assessed by measuring the correlation between the GFP signals prior to, and after 60 min of fixation with either PFA or glyoxal. The graph shows the correlation of the GFP signals pre and post fixation. N = 13-51 cellular ROIs per condition. Scale bar = 10 µm. *p < 0.05, **p < 0.01.

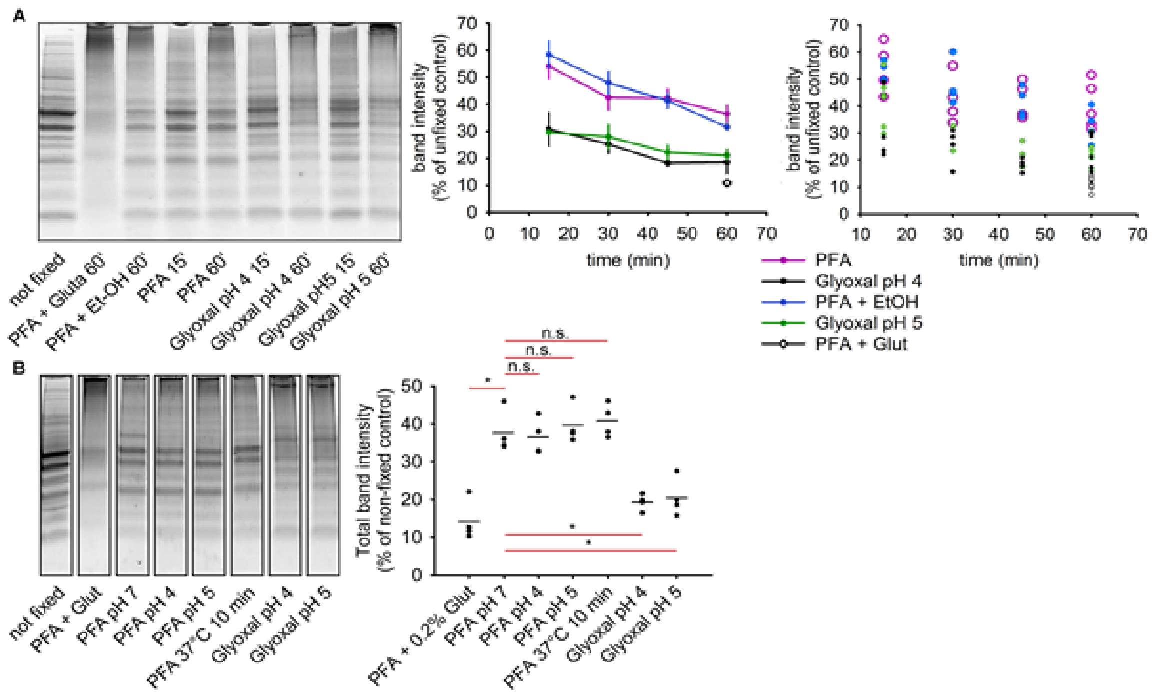

Appendix Figure S7 Comparison of glyoxal fixation with various alternatives of PFA fixation.

A Full polyacrylamide gel from figure 3A. In addition to fixation for 60 minutes with PFA, PFA and glutaraldehyde, glyoxal pH 4, and glyoxal pH 5, which were shown in figure 3, samples were also fixed for 15, 30 and 45 minutes (gels with fixation duration of 30 and 45 min are not shown here). We also fixed samples with PFA + ethanol. The graph shows the remaining intensities of all bands, for all tested time points (either individual data points or mean values with SEM are plotted). This indicates that fixation for shorter durations results in lower fixation strength. Additionally, fixation with PFA + ethanol does not increase the efficiency of fixation, in comparison with PFA alone. N = between 2 and 7 experiments per condition.

B Testing the efficiency of PFA fixation under different alternative conditions (pH 4, pH 5 or at 37°C) in the SDS PAGE based approach (see also figure 3). A quantification of total band intensity left after fixation shows that neither low pH, nor higher temperature increase fixation efficiency significantly, when compared to PFA pH 7 fixation. Glyoxal fixation, which was tested in parallel to these experiments in the same gels, reduced the total band intensity significantly, when compared to these PFA conditions, as in all other SDS PAGE experiments we tested. N = 4 gels in 4 independent experiments. * $p < 0.05$

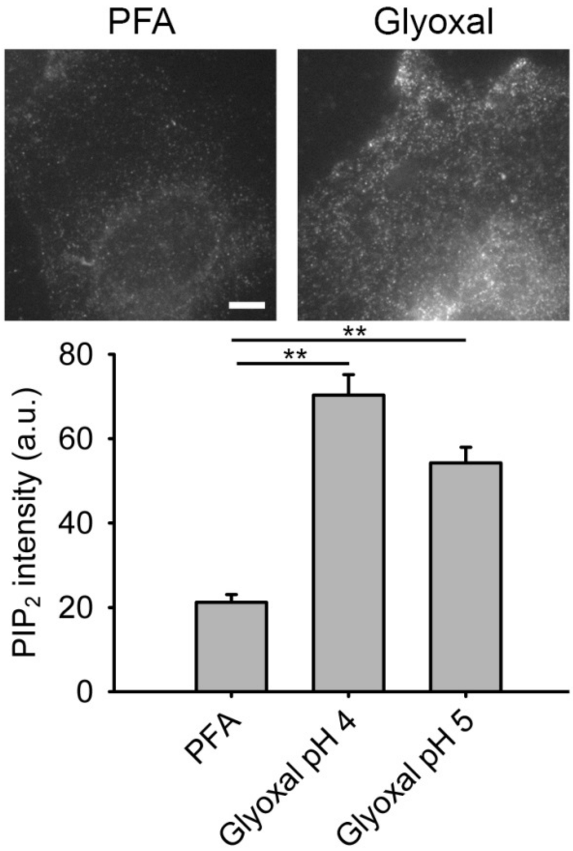

Appendix Figure S8 Fixation of lipids by PFA and glyoxal.

PFA and glyoxal-fixed COS-7 cells were immunostained for Phosphatidylinositol-4,5-bisphosphat (PIP_2), and the fluorescence intensity was measured. Cells that were fixed with glyoxal exhibited significantly brighter stainings than PFA-fixed cells. N = 18-35 cells. Scale bar = 5 µm. **p < 0.01.

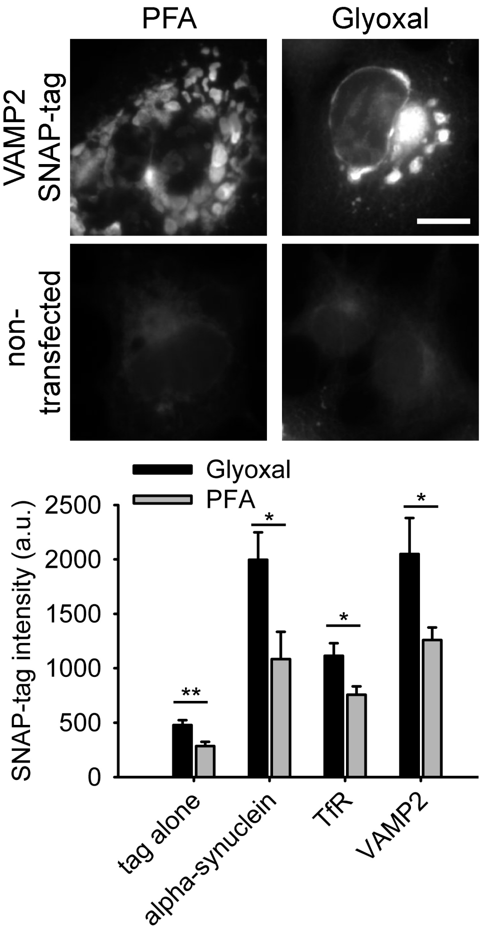

Appendix Figure S9 Preservation of enzymatic activity after PFA and glyoxal fixation.

HeLa cells were transfected with the enzymatic labeling tag SNAP-tag, either alone or linked to three different cellular proteins. After fixation the maintenance of the enzymatic activity was tested by applying a fluorophore that is covalently linked to the tag by its own enzymatic reaction. All tested SNAP-tag variants show significantly higher labeling for glyoxal-fixed cells compared to PFA-fixed cells. Non-transfected cells show no substantial fluorescence. N = 10-14 images analyzed per condition (each containing multiple cells). Scale bar = 2 µm. *$p < 0.05$, **$p < 0.01$.

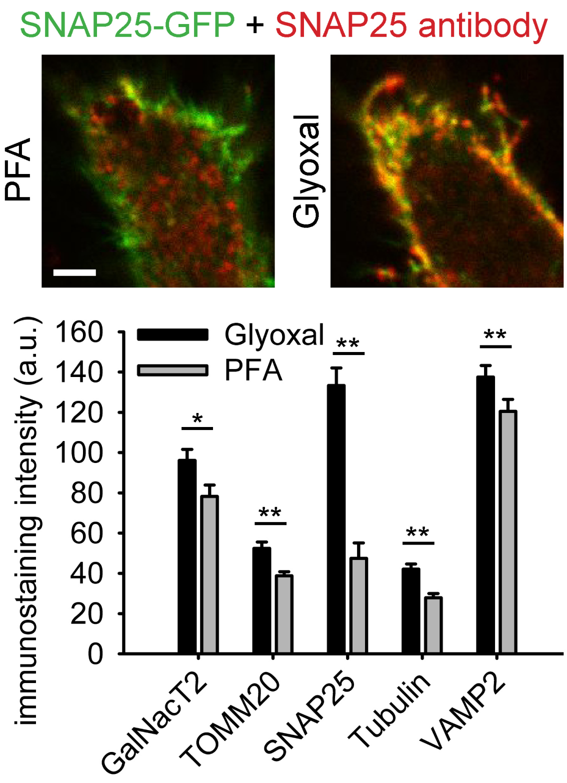

Appendix Figure S10 Immunostaining efficiency after PFA and glyoxal fixation.

COS-7, HeLa or BHK cells overexpressing different GFP-tagged proteins were fixed and immunostained, and the fluorescence intensity of the antibody staining was measured. Immunostaining intensities for all targets were significantly higher in glyoxal-fixed cells than in PFA-fixed cells. N = 5-330 cellular ROIs per condition. Scale bar = 2 µm. *$p < 0.05$, **$p < 0.01$.

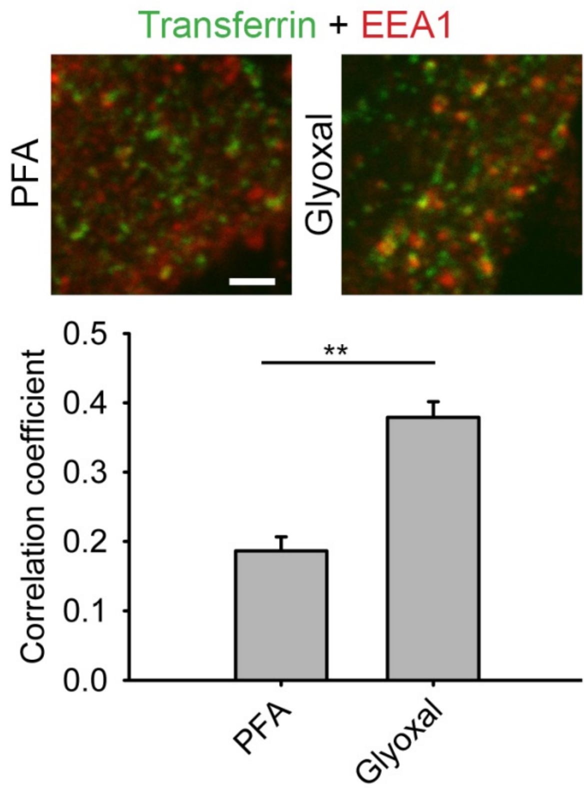

Appendix Figure S11 Preservation and antibody recognition of cellular targets fixed by PFA and glyoxal.

HeLa cells that took up fluorescently-labeled transferrin were immunostained for the endosomal marker EEA1. The correlation of transferrin, which should be taken up into endosomes, to the immunostained EEA1 is significantly higher for glyoxal-fixed cells than for PFA-fixed cells. N = 15 (PFA) and 29 (glyoxal) ROIs analyzed. Scale bar = 2 µm. **p < 0.01.

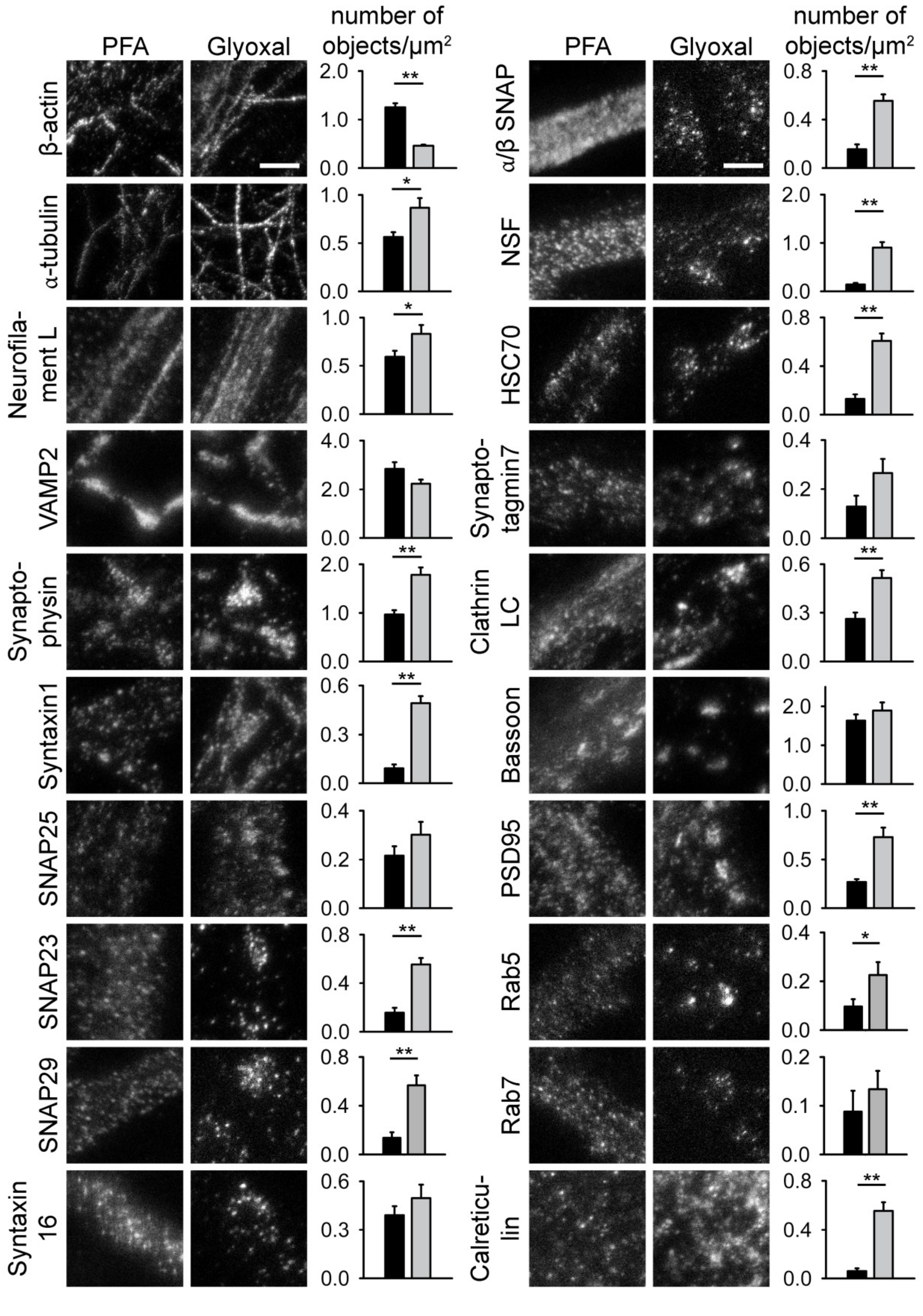

Appendix Figure S12 Super-resolution imaging of 20 immunostained proteins in cultured neurons after fixation with PFA or glyoxal.

Typical images of stained hippocampal neurons are shown, after glyoxal or PFA fixation. The bar graphs indicate the number of organelle-like structures that an experienced user could detect, per stained surface area (black bars = PFA, grey bars = glyoxal). Significantly more objects were identified in glyoxal than in PFA fixed samples for the following proteins: α-tubulin, neurofilament L, synaptophysin, syntaxin 1, SNAP23, SNAP29, α/β SNAP, NSF, HSC70, clathrin light chain, PSD95,

rab5 and calreticulin; N = 7-15. For β-actin, significantly more structures were found in PFA-fixed cells, since the actin filaments appear more fragmented after this treatment, which increases the number of observed strands (see analysis in Appendix Figure S13). Scale bar = 2.5 μm (for β-actin and α-tubulin) and 1 μm (for the rest). * $p < 0.05$, ** $p < 0.01$. All experiments were analyzed in a blind fashion (see Methods for details).

In detail, we analyzed several types of proteins. We started with immunostainings for β-actin, α-tubulin, and neurofilament L, which are expected to form linear (filamentous) structures. Significantly more microtubules and neurofilaments could be detected after glyoxal fixation. In contrast, the number of actin filaments was significantly higher after PFA fixation, due to their fragmentation (analyzed in Appendix Fig. S13). We then analyzed two markers of synaptic vesicles, VAMP2 and synaptophysin. The vesicles form clusters in synaptic boutons. The same number of boutons could be observed in VAMP2 immunostainings, for both glyoxal and PFA fixation. For synaptophysin, however, significantly more boutons could be identified after glyoxal fixation. We next focused on five SNARE proteins, which are present in the synaptic plasma membrane (syntaxin 1, SNAP25), in other sites of the neuronal plasma membrane (SNAP23, SNAP29), or in endosomes (syntaxin 16). SNARE proteins form domains in the membranes (see for example (Bethani *et al*, 2007; Lang & Rizzoli, 2010)). We observed far more domains of syntaxin 1 after glyoxal fixation. Similar, albeit less pronounced, effects were observed for SNAP23 and SNAP29. No significant differences were observed for syntaxin 16 and SNAP25. Several soluble and/or organelle-attached proteins also provided more detailed (less uniform) images after glyoxal fixation, in which significantly more organelle-like structures could be detected (α/β-SNAP, NSF, Hsc70, clathrin, Rab5, and Rab7). The number of observed organelle-like objects were lower in PFA samples for several targets that are anchored to the membrane or to other cellular structures, including syntaxin 1, as mentioned above, and also the ER marker calreticulin and the active zone protein PSD95. Some such targets were detected equally well after both PFA or glyoxal fixation, including synaptotagmin 7, which is associated to vesicles, or the active zone component Bassoon.

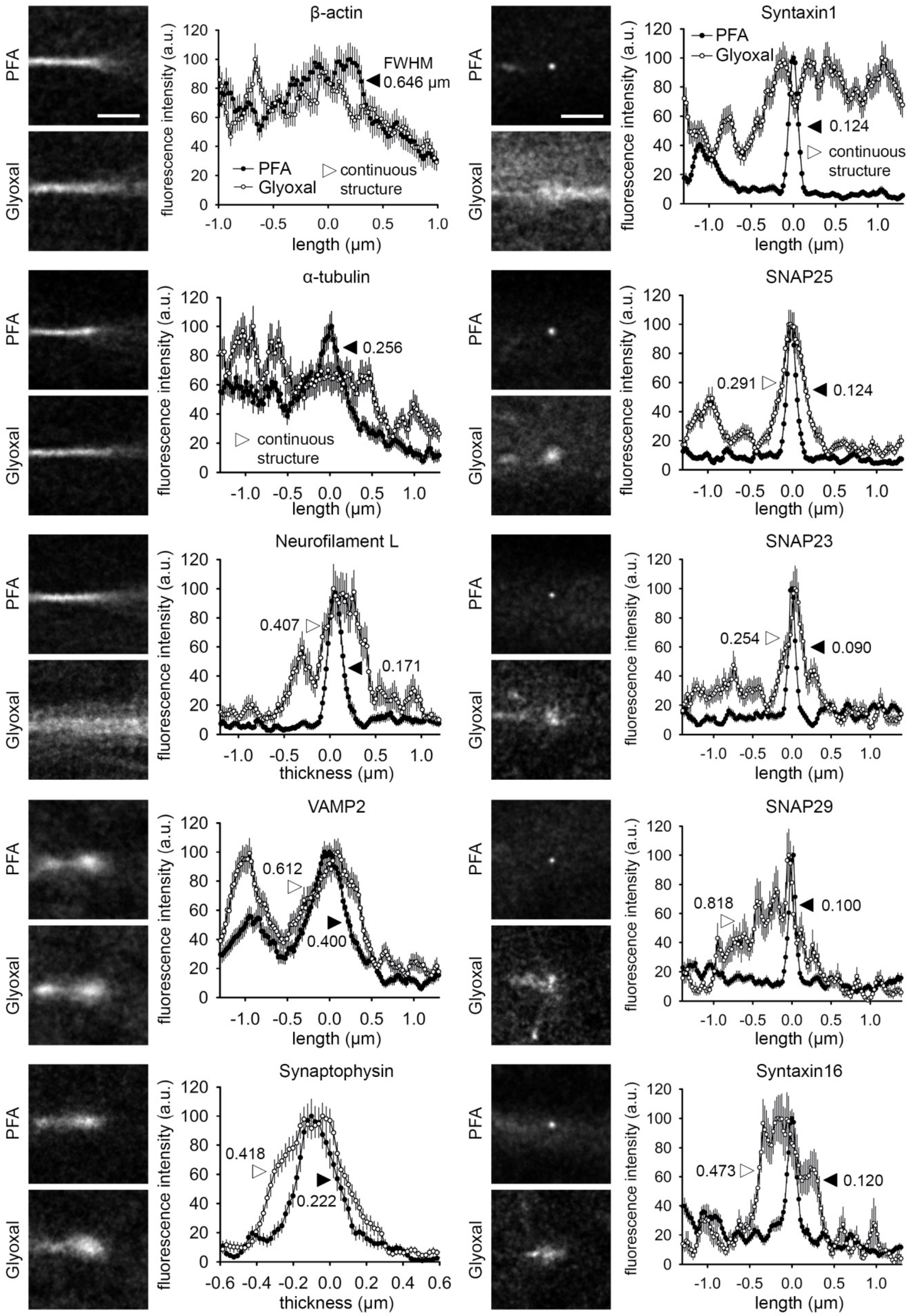

Appendix Figure S13 Ten pairs of average objects (structures) from neurons immunostained after PFA or glyoxal fixation.

To further analyze the difference between the glyoxal- and PFA-fixed samples, we created average objects, or average target structures, by overlaying 100 typical objects for every staining from

Appendix Fig. S12 (again in a blind fashion). The objects were all centered, and were rotated to create a maximal alignment. This procedure is used in super-resolution microscopy to reveal aspects of the average object that may be missed by the visual investigation of only single objects (see for example Löschberger *et al*, 2012; Revelo *et al*, 2014; Wilhelm *et al*, 2014). The typical objects were aligned and averaged, and the resulting averages are shown in the images in Appendix Fig. S13 and S14, in the same order as in the two columns of Appendix Fig. S12. 10 target proteins are included in each of these two figures. Vertical or horizontal line scans indicate the length of the objects, with the exception of neurofilament L, and synaptophysin, for which the thickness of the objects is indicated. The arrowheads (black = PFA, white = glyoxal) and the numbers indicate the size of the objects, in the form of the full width at half maximum (FWHM) of the linescans (in µm). For some graphs the FWHM could not be measured, since there was no prominent single peak present, meaning that the objects consisted of continuous structures. This is noted on the respective graphs, as in the case of syntaxin 1, which is an abundant and widely distributed protein, which is expected to be present in large neuronal areas. The error bars show the SEMs of the line scans (n = 100 objects). Scale bar = 1 µm.

A simple overall conclusion is that the average object after PFA fixation is represented by a spot of ~70 to 120 nm in diameter for 14 of the proteins: syntaxin 1, SNAP25, SNAP23, SNAP29, syntaxin 16, α/β-SNAP, NSF, HSC70, synaptotagmin 7, clathrin, PSD95, Rab5, Rab7, and calreticulin. Such small objects overlap in size with spots created by clusters composed of one primary antibody and the secondary antibodies that bind it (in our hands, at the resolution of the STED microscope used here (Opazo et al., 2010); 2-3 secondary antibodies bind each primary in our experiments (Opazo et al., 2012)). For all of these proteins larger average objects were found after glyoxal fixation, which are too large to be merely single antibody spots. For the remaining 6 target proteins (β-actin, α-tubulin, neurofilament L, VAMP2, synaptophysin, and Bassoon), PFA revealed objects that were similar to those observed in the glyoxal-fixed samples. Actin filaments and microtubuli, however, were substantially shorter after PFA fixation, which implies that they became fragmented during fixation. Neurofilaments were far thinner after PFA fixation than after glyoxal fixation. Synaptic boutons were revealed by VAMP2 or synaptophysin antibodies after both PFA and glyoxal fixation, as mentioned in the previous section, but they were shorter and/or thinner after PFA fixation, suggesting that fewer epitopes were revealed. Finally, active zones were recognizable in the staining for the marker Bassoon, for both PFA and glyoxal, but they appeared larger after glyoxal fixation (Appendix Fig. S14).

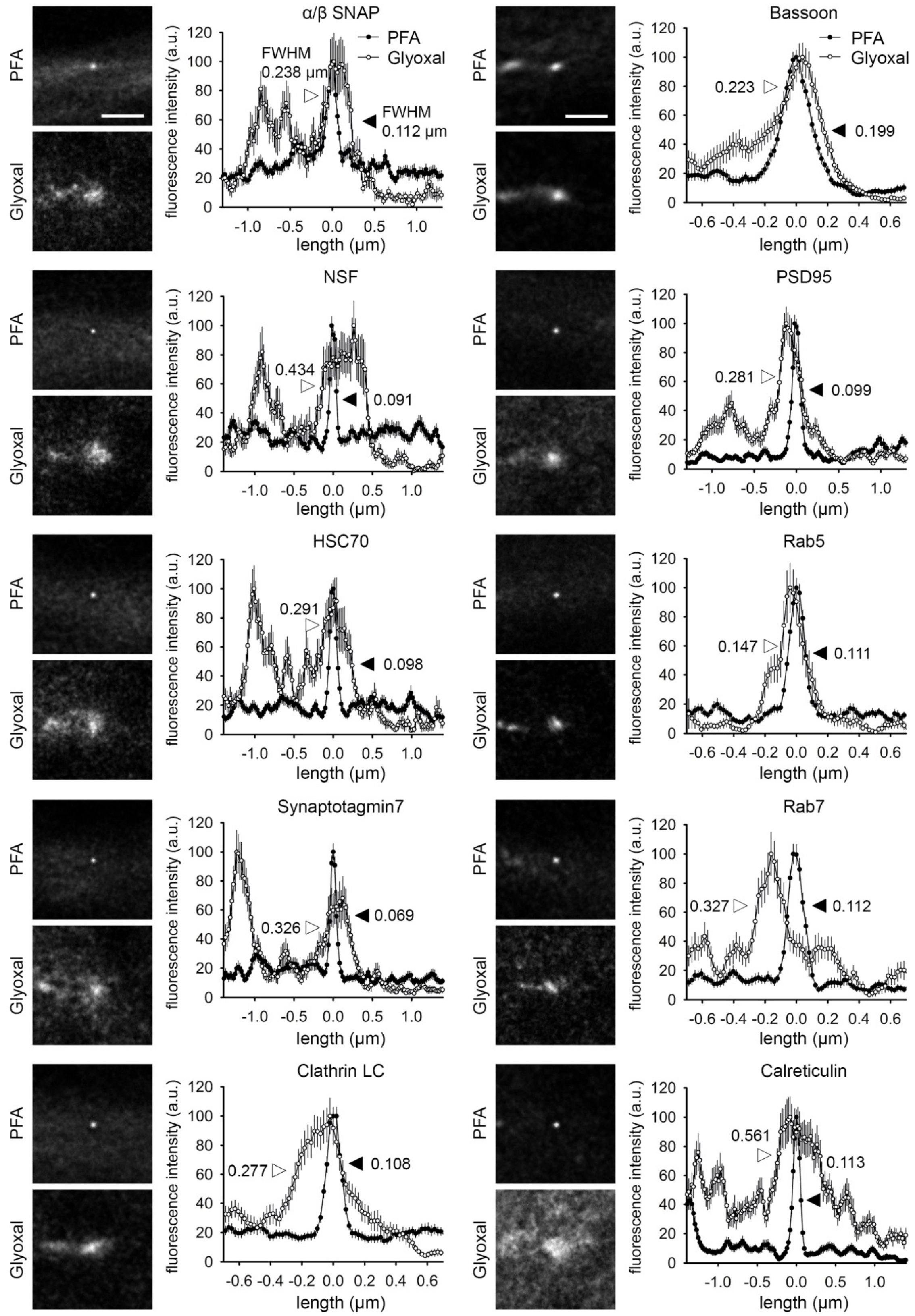

Appendix Figure S14 Ten additional pairs of averaged objects from neurons immunostained after PFA or glyoxal fixation (continuation of Appendix Figure S13). The analysis was performed exactly as in Appendix Figure S13. Arrowheads (black = PFA, white = glyoxal) and numbers indicate the FWHM of the linescans (in µm), as in Appendix Figure S13. Scale bar = 1 µm.

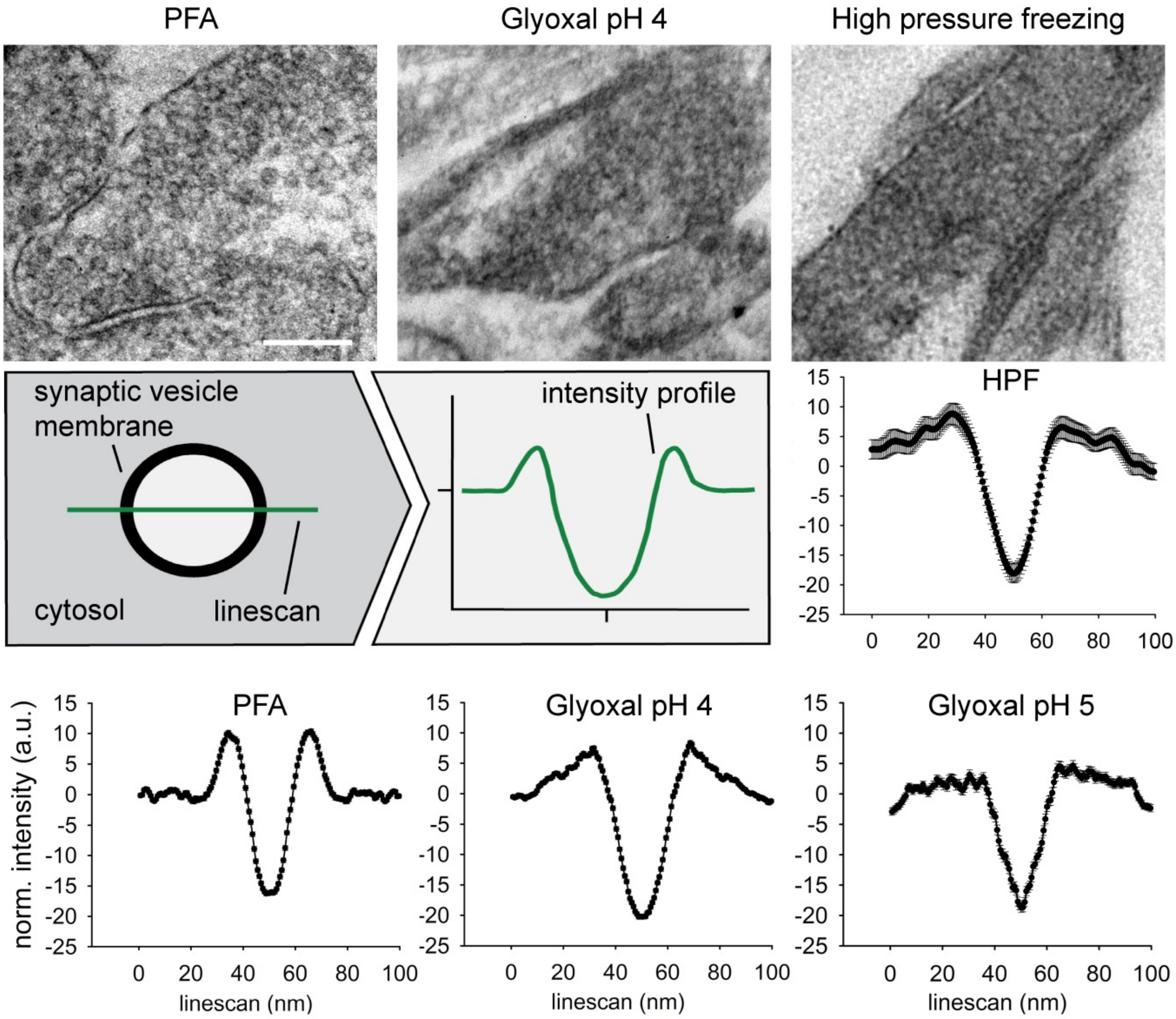

Appendix Figure S15 Electron microscopy of PFA- or glyoxal-fixed hippocampal neurons.

Representative images of synaptic boutons fixed with PFA or glyoxal, in cultured hippocampal neurons, are shown. For comparison, a representative image of cultured hippocampal neurons fixed via high-pressure freezing (HPF) and freeze-substitution is shown in the right panel. The graphs indicate average line scans performed through synaptic vesicles of the fixed neurons (see cartoon), ± SEM (note that the SEMs are almost as small as the graph dots for the chemical fixed samples). The intensity was normalized to the background intensity of the images, outside of the vesicles. The line scan has a minimum in the center, corresponding to the center of the synaptic vesicle lumen, which is devoid of proteins. The vesicle membrane is clearly visible, and is significantly more electron-dense than the baseline outside the vesicles in the PFA-fixed samples, suggesting that the cytosol contains fewer proteins than the membrane of the vesicles in this condition. This effect is far less pronounced in glyoxal-fixed samples and the HPF-fixed sample, indicating that more cytosolic proteins are retained around the vesicles. This effect also explains the general lack of contrast in the glyoxal-fixed sample, in which vesicles are more difficult to detect. Superficially, images of glyoxal-fixed boutons resemble boutons fixed by high-pressure freezing more than they resemble PFA-fixed boutons: the demarcation of the vesicles is more difficult, due to the large amount of cytosolic proteins that are present around and between the vesicles. Scale bar = 200 nm. N = 1100-4900 vesicles, from 2-4 independent experiments for each chemical fixation procedure and 782 vesicles for the HPF-fixation.

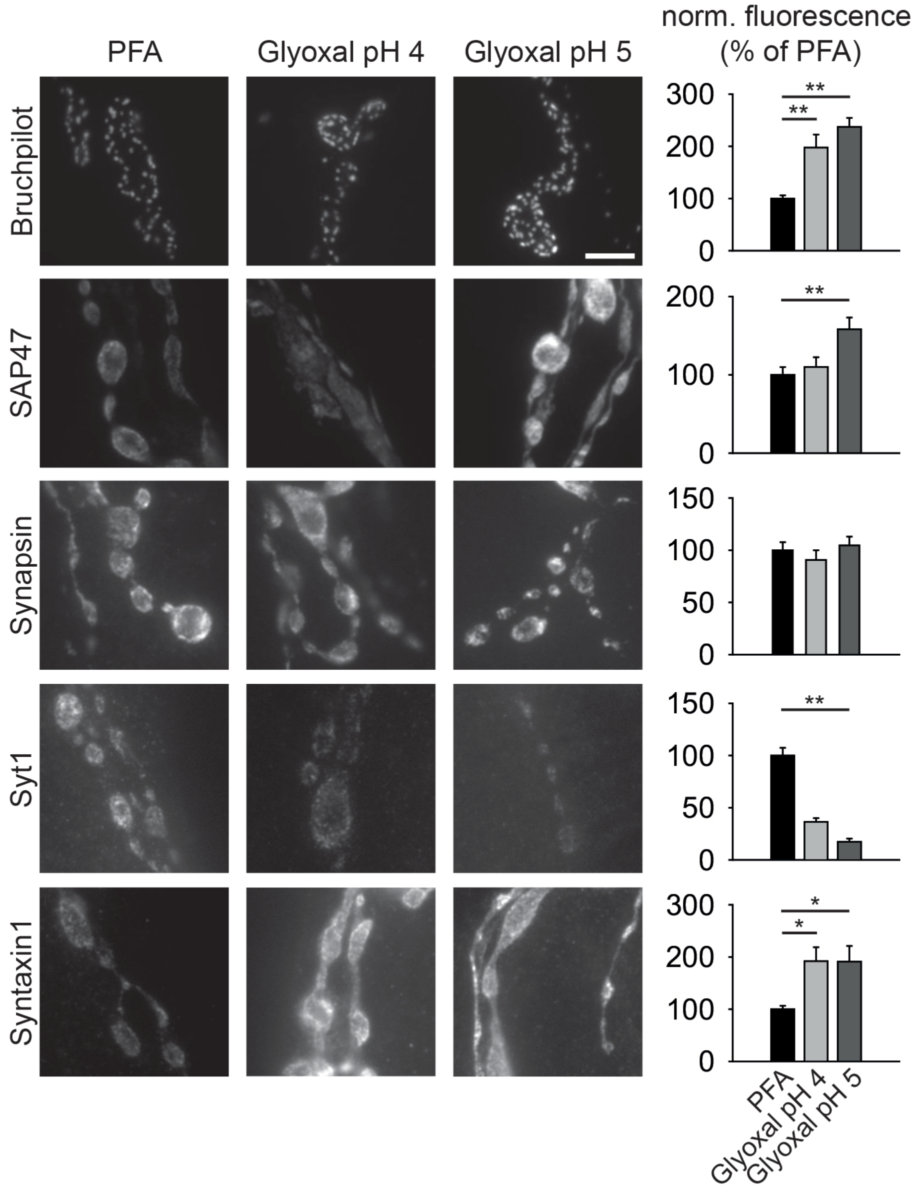

Appendix Figure S16 Glyoxal fixation in tissue from *Drosophila* larvae.

Fluorescence images of *Drosophila* neuromuscular junctions, immunostained for various neuronal proteins, are shown. The graphs indicate the average fluorescence intensity, corrected for background. Glyoxal pH 4 and pH 5 fixations provide significantly higher fluorescence signals, in comparison to PFA, for bruchpilot and syntaxin 1. Glyoxal pH 5 provides a significantly higher signal for SAP47. PFA fixation gives a significantly higher signal for synaptotagmin 1 (Syt1), in comparison to the glyoxal fixation. N = between 15 and 20 images analyzed per protein and fixation condition, in 2 independent experiments. Scale bar = 5 µm. $^*p < 0.05$, $^{**}p < 0.01$.

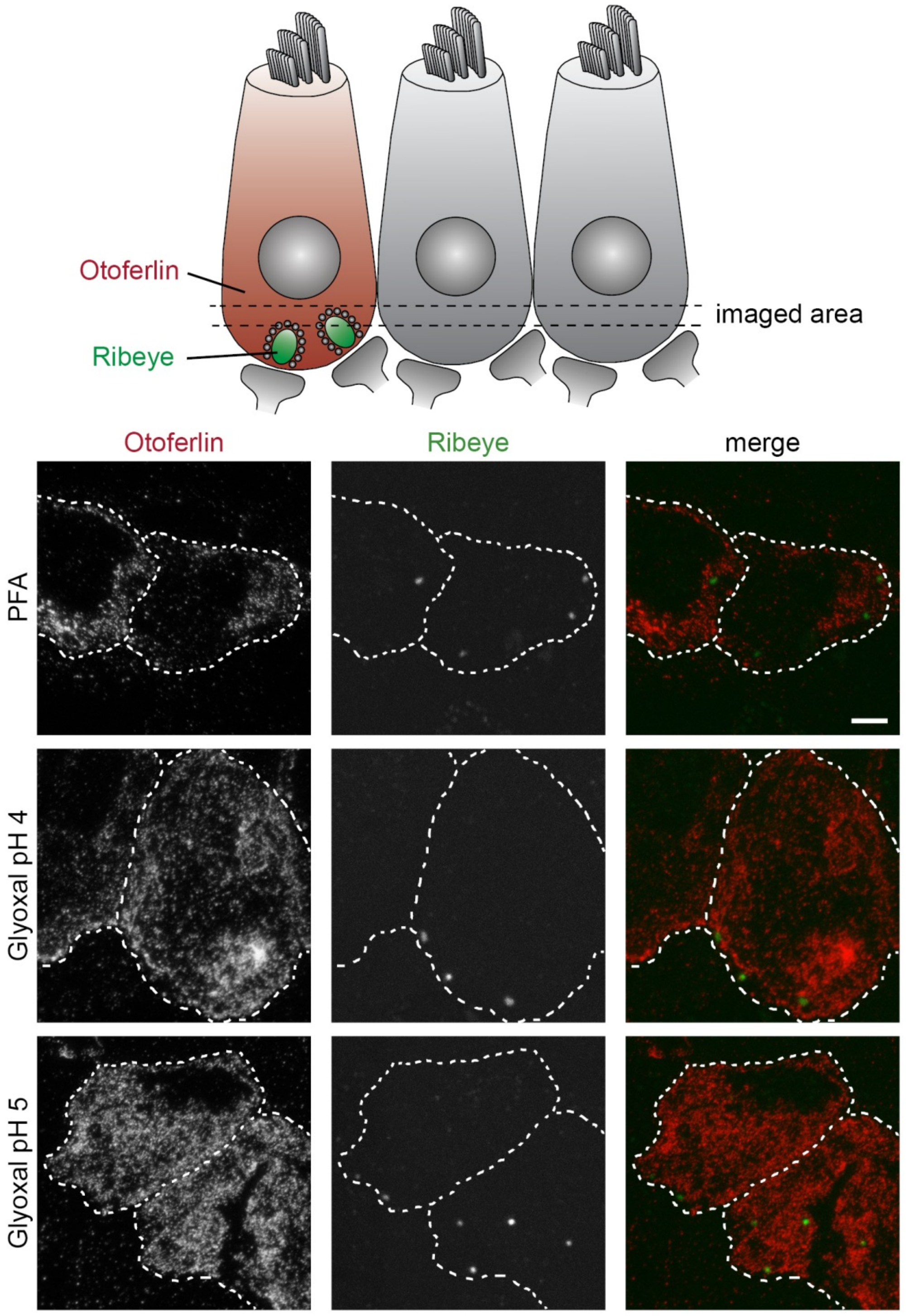

Appendix Figure S17 Glyoxal fixation in mammalian tissue: mouse inner hair cells.

Inner hair cells of the auditiory system (Revelo *et al*, 2014) were immunostainined for the calcium sensor protein otoferlin (Atto647N, red), and for the ribbon synapse protein ribeye (Cy2, green). The overall morphology and antigenicity is preserved in glyoxal-fixed samples, at least as well as in PFA-fixed samples. Scale bar = 2 µm.

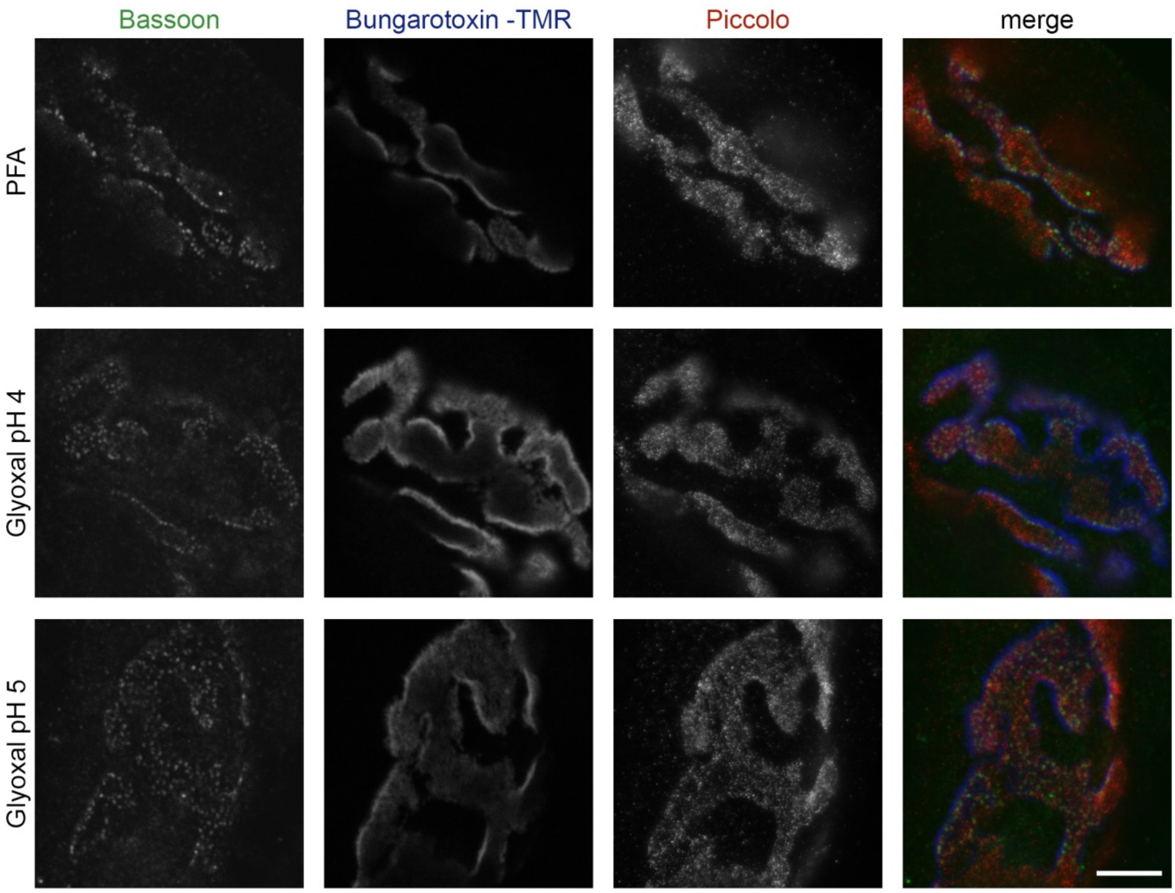

Appendix Figure S18 Glyoxal fixation in mammalian tissue: mouse neuromuscular junctions.

Synapses of the *levator auris longus* muscle were immunostained for bassoon (Cy2, green) and piccolo (Atto647N, red). Additionally, acetylcholine receptors were stained with tetramethylrhodamine-labeled bungarotoxin (blue). As in Supplementary Fig. 10, the overall morphology and antigenicity is preserved in glyoxal-fixed samples. Scale bar = 5 µm.

Appendix Table 1: Qualitative analysis of different fixation conditions. Hippocampal neurons were fixed for 1 hour with the fixatives indicated in the first column, and were then imaged in presence of 1.5 µM FM 1-43, which reveals all membranes. Scale bar, 5 µm. + and – indicate the quality of the fixation regarding possible fragmentation and swelling

Fixative	Comment	Quality	Image
4% PFA + 0.2% Glut	No fragmentation, but some swelling (note large round objects)	+/-	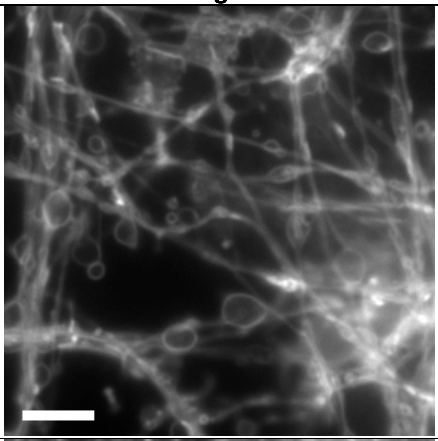
3% Glyoxal + 20% Ethanol, pH 3	Some fragmentation (note the small objects arranged in line), no swelling	-/+	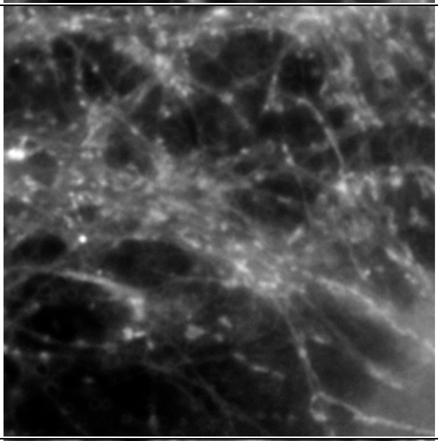
3% Glyoxal + 20% Ethanol, pH 4	No fragmentation, no swelling	+/+	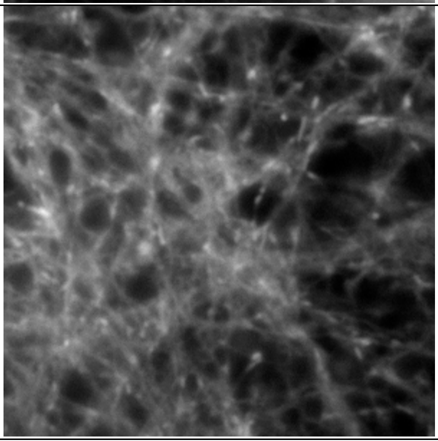

3%<br>Glyoxal, pH 4	No fragmentation, but some swelling (especially visible in the image center)	+/-	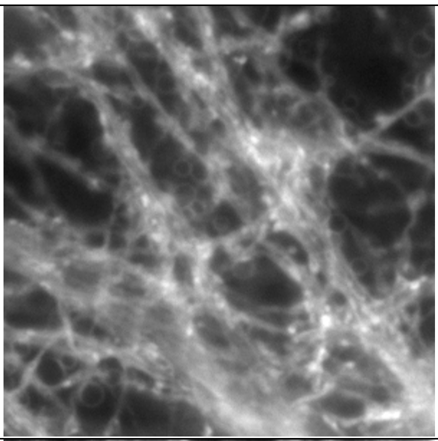
3% Glyoxal + 20% Ethanol, pH 5	No fragmentation, very limited swelling	+/+	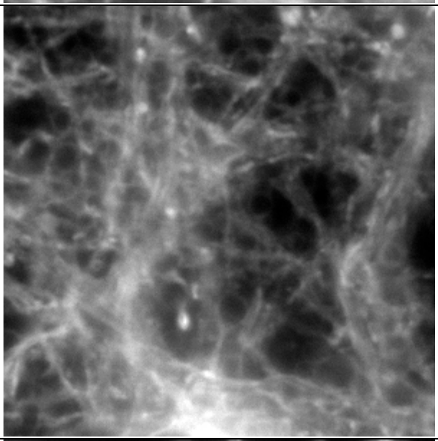
3% Glyoxal, pH 5	No fragmentation, but very strong swelling	+/--	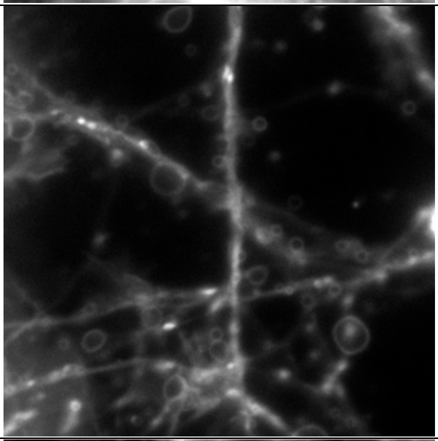
3% Glyoxal + 20 % Ethanol, pH 6	No fragmentation, but very strong swelling	+/--	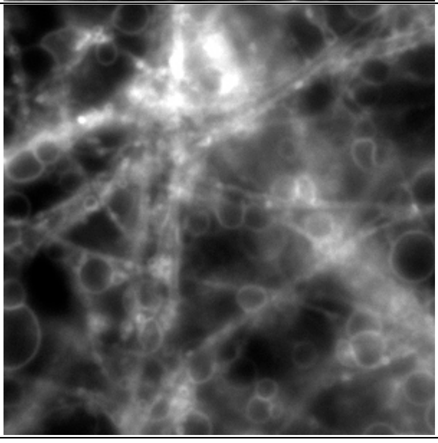

PFA, pH 4	Some fragmentation, some swelling	-/-	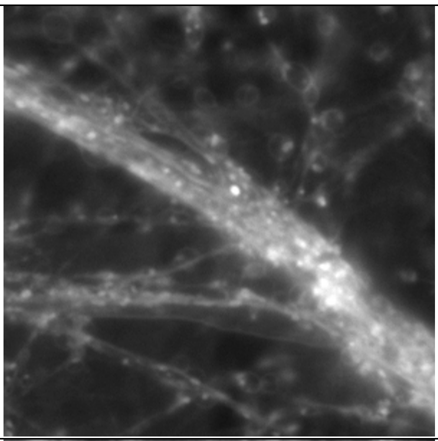
PFA + 20 % Ethanol, pH 4	Some fragmentation, limited swelling	-/-	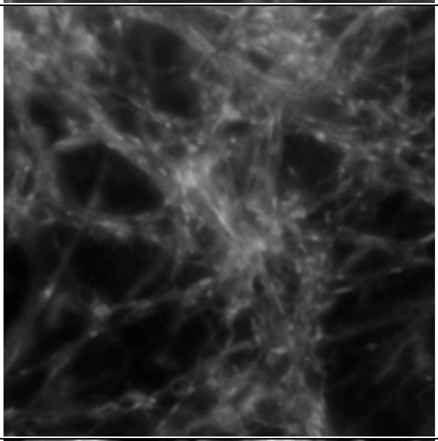
PFA, pH 5	Some fragmentation, some swelling	-/-	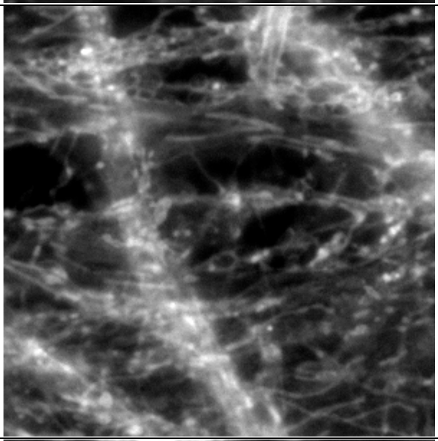
PFA + 20 % Ethanol, pH 5	Limited fragmentation, strong swelling	+/--	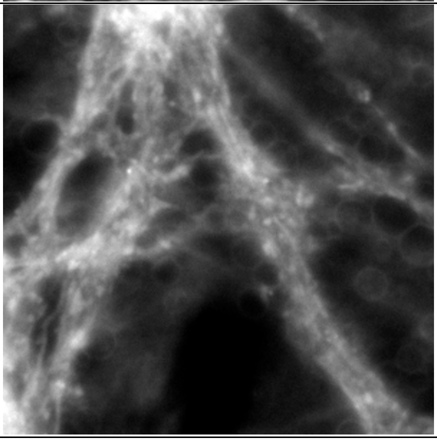

PFA, pH 7	Visible fragmentation, very strong swelling	-/--	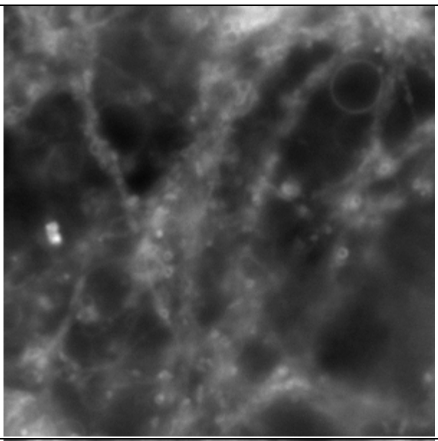
PFA + 20 % Ethanol, pH 7	Visible fragmentation, strong swelling	-/-	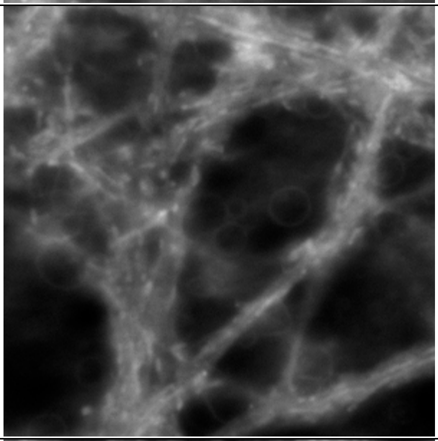
PFA, 37°C, 10 minutes	Very strong fragmentation, very strong swelling	--/--	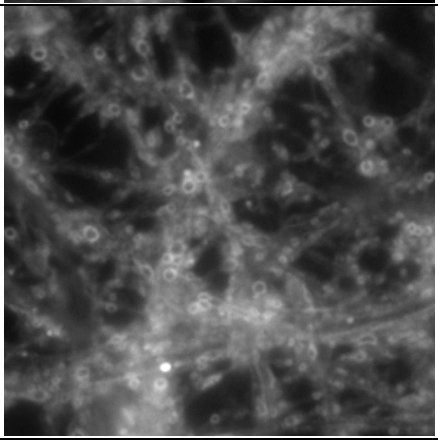

Methods of collaborating laboratories

Ed Boyden

Immunocytoschemistry of HeLa cells (Figure 5)
HeLa Cells were fixed with either 4% PFA of glyoxal for 10 min at room temperature. Afterwards, cells were quenched for 5 min in PBS and 100 mM glycine, following 10 min washing in PBS. Subsequently, the cells were incubated in the primary antibody (NUP160; abcam ab74147) for one hour at room temperature. The cells have been washed 20 min in PBS prior to the incubation in Atto647F coupled secondary antibody (Sigma Aldrich) for one hour at room temperature. Both antibody incubations have been done in blocking buffer (5% normal donkey serum, 0.25% Triton X-100 in PBS). After further washing in PBS for 20 min the cells were imaged with a spinning disk confocal microscope.

Rory Duncan

Immunocytochemistry of AtT20 cells (Figure 6)

For Syntaxin1, SNAP25 and LC3B immunostaining, AtT20 cells were cultured in duplicate on PDL-coated coverslips and fixed with 4% PFA or glyoxal pH 5 by 60 minute incubation at room temperature. Autophagy was induced in cells intended for LC3B staining by 60 minute treatment with 160 nM rapamycin immediately prior to fixation. Following fixation, cells were quenched with 50 mM NH_4Cl for 20 min and permeabilized by 4 minute incubation in PBS supplemented with 0.5% (v/v) Triton X-100. Epitope blocking was subsequently achieved by 15 minute incubation in blocking buffer (0.5% (w/v) fish skin gelatin in PBS). Samples were immunolabeled for three hours at room temperature with the primary antibodies anti-Syx1 [HPC-1], anti-SNAP25 [SMI 81] or LC3B diluted in blocking buffer as detailed in Appendix Table 2. Cells were then washed for 30 mins in blocking buffer and incubated for 60 minutes in blocking buffer supplemented with 1:1000 Alexa 488 conjugated anti-rabbit IgG or anti-mouse IgG as appropriate. Samples were mounted with Mowiol after a further 30 min wash step. Samples were imaged on a Leica SP5 confocal laser scanning microscope using a HCX PL Apo 100X oil immersion objective lens. The pinhole was set to 1 Airy unit and images were acquired with a 1024x1024 pixel layout using a 0.04 µm pixel size.

Appendix Table 2: Primary and secondary antibodies used for the immunostaining of AtT20 cells

**Primary antibody**	**Host species**	**Company**	**Dilution**
Syx1 [HPC-1]	Mouse	Abcam ab3265	1:1000
SNAP25 [SMI 81]	Mouse	Abcam ab24737	1:1000
LC3B	Rabbit	Novus Biologicals NB600-1384	1:500
**Secondary antibody**	**Host species**	**Company**	**Dilution**
Anti-rabbit IgG (Alexa Fluor 488)	Goat	Fisher Scientific (10082502)	1:1000
Anti-mouse IgG (Alexa Fluor 488)	Goat	Fisher Scientific (10328172)	1:1000

Elisa D'Este/Stefan Hell

Neuronal cell culture and immunocytochemistry (Figure 7)

Cultures of hippocampal neurons were prepared from Wistar rats of mixed sex at postnatal day P0–P1, in accordance with Animal Welfare Law of the Federal Republic of Germany (Tierschutzgesetz der Bundesrepublik Deutschland, TierSchG) and the Regulation about animals used in experiments (1st August 2013, Tierschutzversuchsverordnung). For the procedure of sacrificing rodents for subsequent preparation of any tissue, all regulations give in §4 TierSchG are followed. Since sacrificing of animals is not an experiment on animals according to §7 Abs. 2 Satz 3 TierSchG, no specific authorization or notification is required. Cells were plated on coverslips coated with 100 µg/ml polyornithine (Sigma-Aldrich #P3655) and 1 µg/ml laminin (BD Bioscience #354232). Neuronal cultures were maintained in Neurobasal medium (Gibco #21103049) supplemented with 2% B27 serum-free supplement (Gibco #17504044), 2 mM L-glutamine (Gibco #25030) and penicillin/streptomycin (100 units/ml and 100 µg/ml, respectively, BiochromAG A2213). On the day after plating, 5 µM cytosine β-D-arabinofuranoside (Sigma #C1768) was added to the cultures.

For immunostainings, cells were washed in PBS and fixed for 20-25 min at room temperature with glyoxal pH 5 (except for staining of neurofilament and spectrin pH 4 was used) or PFA or PFA with 0.2 % glutaraldehyde in PHEM buffer (60 mM PIPES, 25 mM HEPES, 10 mM EGTA, 2 mM MgCl2; pH 6.9). Cells were quenched with ammonium chloride and glycine (100 mM each) for 5 min, were permeabilized with 0.1% Triton X-100 for another 5 min, and blocked in PBS supplemented with 1%

BSA for 30 min. Both primary and secondary antibody incubations were performed in PBS for 1 hour at room temperature. Phalloidin incubation was performed together with secondary antibodies. Samples were mounted in Mowiol supplemented with DABCO.

Reagents and antibodies

The antibodies and reagents used are: anti-betaIII tubulin (Tuj1) (Biolegend, 1:400 dilution), anti-betaII spectrin (BD Biosciences, 1:200 dilution); anti-Ankyrin G (SantaCruz, 1:50); anti-neurofilament L (Synaptic Systems, 1:400); anti-pan-Nav (Sigma-Aldrich, 1:100); anti-Kv7.2 (Synaptic Systems, 1:200); phalloidin-STAR635 (Abberior). Sheep anti-mouse (Dianova) and goat anti-rabbit (Dianova) were labeled with STAR635P or START580 (Abberior)

Imaging was performed on a two-color Abberior STED 775 QUAD scanning microscope (Abberior Instruments GmbH, Göttingen, Germany) equipped with 561 nm and 640 nm pulsed excitation lasers, a pulsed 775 nm STED laser, and a 100x oil immersion objective lens (NA 1.4).

Marcel Lauterbach

Immunhistochemistry of *Sepia officinalis* skin samples (Figure 8)

The experiment was conducted on adult male *Sepia officinalis* with a mantle length of 10 cm. Animals were reared in a closed seawater system with salinity of 33‰ at 20°C. To harvest skin samples, animals were anesthetized in an isotonic solution prepared by mixing a 7.5% (w/v) $MgCl_2$* $6H_2O$ in deionized water solution with an equal volume of seawater. Animals were then decapitated and samples of dorsal skin and fin were excised and fixed overnight at 4°C in either 3 % glyoxal pH 5 or 4% PFA. The following procedures were the same for both fixation techniques and performed in parallel. After cryoprotection in 30% (w/v) sucrose in PBS for 2 days at 4°C the tissue was embedded in Tissue-Tek O.C.T. (Sakura Finetec Europe B. V., Alphen aan den Rijn, The Netherlands) and sectioned at 100 µm thickness.

Prior to permeabilization in acetone for 15 min at -20°C, the sections were washed twice for 10 min in PBS. Samples were blocked for 2 hours at room temperature in a freshly prepared solution containing 2% Normal Goat Serum, 1% Bovine Serum Albumin, 0.2% Triton X-100, 0.1% cold water fish gelatin, 0.3M glycine and 0.05% thimerosal. The samples were incubated for 18 hours at 4 °C with the primary antibody diluted in blocking solution. The antibody used was a rabbit polyclonal antibody against FMRFamide (1:500, ImmunoStar Inc, Hudson, Wisconsin, USA, Cat. No. 20091, RRID: AB_572232). After 6 x 10 min washing in PBS containing 0.2% Tween 20 (PBST) the samples were incubated with the secondary antibody (Alexa 488 anti-rabbit (Thermo Fisher Scientific, Waltham, Massachusetts, USA, Cat. No. A-11008). The sections were washed 6 x 15 min in PBST and mounted in Fluorescence Mounting Medium (Dako, Agilent Technologies, Santa Clara, California, USA). Confocal fluorescence images were recorded at an LSM 880 microscope (Zeiss, Oberkochen, Germany) with a 63x NA 1.2 Oil immersion objective.

Stephan E. Lehnart

Isolation and immunocytochemistry of murine ventricular myocytes (Figure 9)

Ventricular myocytes (VM) of 12 week old C57Bl/6N mice were isolated according to previously published protocols (Wagner *et al*, 2012, Wagner *et al*, 2014). In short, hearts were attached to a modified Langendorff perfusion system and digested by perfusion through the aorta using collagenase type II (Worthington).

Freshly isolated VM were immediately plated on laminin-coated coverslips. After 30 min of plating, VM were either fixed with 4% PFA, pH 7.4 or with 3% glyoxal, pH 5 for 10 min at room temperature. After fixation, VM were washed with blocking buffer (0.2% Triton X-100, 10% bovine calf serum in PBS) once. Primary antibodies were diluted in blocking buffer and VM were incubated with the appropriate antibody dilution (Appendix Table 3) over night at 4°C. Afterwards, VM were washed with blocking buffer three times, secondary antibodies were diluted in blocking buffer and VM were incubated with the appropriate secondary antibody dilution (Appendix Table 3) over night at 4°C. After three washing

steps with PBS, pH 7.4, VM were mounted using ProLong Gold Antifade Mountant (molecular probes via Thermo Fisher).
Immunostained murine VM were imaged using a TCS SP8 STED microscope (Leica) and a 100x oil objective (1.4 NA). Abberior STAR 635P was exited at a wavelength of 635 nm and fluorescence was detected between 650 nm and 700 nm. STED imaging was performed with a 775 nm depletion laser and gating between 0.5 ns and 6 ns. All images were acquired with a pixel size of 23 nm x 23 nm and a scanning speed of 600 Hz (pixel dwell time 0.4 µs).

Appendix Table 3: Antibodies used for immunostaining of murine VM

**Primary antibody**	**Host species**	**Company**	**Dilution**
caveolin-3	rabbit	Abcam (#ab2912)	1:500
ryanodine receptor type 2	mouse	Thermo Fisher (#Ma3-916)	1:500
**Secondary antibody**	**Host species**	**Company**	**Dilution**
anti-rabbit Abberior STAR635P	goat	Abberior (#2-0012-007-2)	1:1000
anti-mouse Abberior STAR635P	goat	Abberior (#2-0002-007-5)	1:1000

Tobias Moser

Immunohistochemistry of auditory inner hair cells (Figure 10)
Acutely dissected organs of Corti from hearing p14-p16 mice were fixed (either 10 min in ice-cold fixative or 1 h on ice) using either 4% PFA or glyoxal at pH 5.0, and were then permeabilized and blocked with goat serum dilution buffer (GSDB) containing 16% normal goat serum, 450 mM NaCl, 0.3% Triton X-100 and 20 mM phosphate buffer at pH 7.4. Subsequently, the following primary antibodies were applied in GSDB over night at 4°C: mouse monoclonal anti-otoferlin (Abcam; ab#53233), rabbit polyclonal anti-calretinin (Swant; #7697), mouse monoclonal CtBP2 (BD Bioscience; 612044), CaV1.3 (Alomone; ACC-005), Homer1 (Synaptic Systems; 160 002) and mouse monoclonal anti-PSD95 (clone 7E3-1B8; Sigma Aldrich; P246). After extensive washing in PBS, goat anti-mouse Abberior STAR580 and goat anti-rabbit Abberior STAR635p fluorophore-conjugated secondary antibodies (Abberior 2-0002-005-1 and Abberior 2-0012-007-2, respectively) were used for visualization. Finally, immunolabeled organs of Corti were washed repeatedly in PBS prior to mounting with Mowiol. In all cases, the dissections, fixations and immunostaining procedures were performed in parallel and specimens were subsequently imaged during the same imaging session.

Image acquisition was performed in confocal mode on an Abberior Instruments Expert Line STED microscope (based on an Olympus IX83 inverted microscope) running Imspector software, with excitation lasers at 561 and 640 nm and a 1.4 NA UPlanSApo 100x oil immersion objective. Image stacks were acquired with xy pixel sizes of 60 x 60 nm and a z-step size of 200 nm. The image stacks were summed to generate 2D projection images, before analyzing the total staining intensities.

Tiago Outeiro

Cell culture and Transfection
Human neuroglioma cells (H4) were cultured in Opti-MEM I with Glutamax (Life Technologies- Gibco, Carlsbad, CA, USA) supplemented with 10% Fetal Bovine Serum Gold (PAA, Cölbe, Germany) and 1% Penicillin-Streptomycin (PAN, Aidenbach, Germany), at 37 °C, with 5% CO_2. 24h prior to transfection, H4 cells were plated in 12-well plates (Costar, Corning, New York, USA). On the subsequent day, the cells were transfected with the SynT and Synphilin-1 constructs, to induce the formation of aSyn assemblies, or with the Vimentin-O2 construct, using the calcium phosphate method. Briefly, a mix of DNA, H20 and 2.5M calcium chloride was added dropwise to 2× BES-buffered saline solution (50 mM BES, 280 mM NaCl, 1.5 mM $Na_2HPO_4xH_2O$, pH 6.98). The cells were incubated with plasmid-calcium-phosphate coprecipitates for 17h, after which the media were exchanged, and the cells were allowed to recover for 24h before fixation.

Immunocytochemistry (Figure 11)
Cells were fixed with 4% paraformaldehyde or with 3% glyoxal (pH 5) at room temperature (RT) for 10 minutes. The cells were afterwards permeabilized with 0.1% Triton X-100 (SigmaAldrich, St. Louis, MO, USA). The cells transfected with Vimentin-O2 were stained with DAPI and imaged. For the SynT+ Synphilin-1 model, cells were blocked in 1.5% normal goat serum (PAA, Cölbe, Germany)/1xPBS (1.37 M NaCl, 27 mM KCl, 101.4 mM $Na_2HPO_47.H_2O$, 16.7 mM KH_2PO_4), and were then incubated with primary antibody: Syn1 (1:1000, BD Transduction Laboratory, New Jersey, USA) or Vimentin (1:1000, SigmaAldrich, St. Louis, MO, USA) for 3h, and with secondary antibody (Alexa Fluor 555 goat anti rabbit IgG, (Life Technologies- Invitrogen, Carlsbad, CA, USA)) for 30min at RT. Cells were finally stained with DAPI (Life Technologies- Invitrogen, Carlsbad, CA, USA) (1:5000 in DPBS) for 5 min, and were maintained in 1xPBS for imaging.

Imaging with an inverted epifluorescence Olympus IX 71 microscope
All samples were imaged using an Olympus IX 71 epifluorescence microscope, equipped with a 100 W mercury lamp and a F-View II CCD camera (Soft Imaging Systems GmbH). A 60X UPlanApo oil immersion objective (NA 1.35) from Olympus was used. Excitation and emission filter with 562/585 nm was used and image acquisition was performed using the Olympus Cell^P software.

Peter Rehling

Immunocytochemistry of U2OS and HeLa cells (Figure 12)
Cells were grown on coverslips and MitoTracker™ Orange CMTMRos (Thermofisher, M7510) was applied for 20 minutes at 37°C. Cells were washed briefly in PBS and fixed in 4% paraformaldehyde (AppliChem A3813, 0500) or 3% glyoxal for 20 min at 37°C. Cells were washed with PBS, permeabilized with 0.2% Triton-X100 (Roth, 3051.3) for 20 min, washed again and blocked in blocking buffer (1% bovine serum albumin in PBS) for 20 min at room temperature. Subsequently, cells were incubated in home-made primary rabbit antibodies anti-ATP5B, anti-TIM23, anti-COA6 and anti-NDUFA9, diluted 1:200 in PBS for 60 minutes. Following washing in PBS, cells were incubated with secondary antibodies (Alexa Fluor 488 goat anti-rabbit IgG, ThermoFisher Scientific, A-11001) for 30 min. Final washing was followed by mounting in histology mounting medium containing DAPI (Fluoroshield™; Sigma-Aldrich, F6057). Images were taken with a Leica TCS SP5 confocal microscope using a 100X HCX PL APO oil immersion STED objective (NA 1.4). Images were acquired using the Leica LAS AF imaging software, with a pixel size of 60 x 60 nm and a scanning speed of 1000 Hz.

Blanche Schwappach

Immunocytochemistry of HeLa P4 cells (Figure 13)
HeLa P4 cells (Charneau et al., 1994), were obtained from the NIH AIDS Reagent Program and were grown in DMEM (Gibco #41966-029) supplemented with 10% (v/v) FBS (Biochrom #S0615) and 2 mM L-glutamine (Gibco #25030-024) under 5% CO_2 at 37°C. They were tested for contamination by mycoplasma on a regular basis.
For the glucocorticoid receptor stimulation (employed for the glucocorticoid receptor stainings) cells were treated with the corresponding volume of solvent (absolute ethanol) or a stock resulting in a final concentration of 100 nM of dexamethasone (Sigma #D4902) in DMEM for 60 min at 37°C.
For immunofluorescence, cells were fixed with 4% (w/v) PFA (PanReac AppliChem #A3813) in PBS or 3% w/v glyoxal pH 5 solution, for 15 min (cells stained for SGTA and EEA1) or 60 min and then permeabilized with 0.3% Triton X-100/0.05% SDS in PBS for 10 min at room temperature. Samples were blocked with 10% FBS in PBS for 30 min and incubated with primary antibodies (see Appendix Table 4) diluted in blocking buffer overnight at 4°C. Incubation with Alexa Fluor secondary antibodies (Invitrogen) was performed for 60 min at room temperature. The samples were mounted with Mowiol-

DAPI for the confocal microscope or incubated with DAPI (1 µg/mL, Sigma-Aldrich #D9542) in PBS for 10 min for the screening microscope.

Appendix Table 4: primary antibodies used for immunocytochemistry

primary antibody	host species	company	dilution
BAG6	rabbit	custom-made	1:300
APPL1	rabbit	Cell Signaling; 3858	1:200
ATPB	mouse	abcam; ab14730	1:400
Calnexin	mouse	BD transduction; 610547	1:200
Caveolin-1	rabbit	Cell Signaling; 3267	1:200
EEA1	mouse	BD transduction; 610456	1:200
Emerin	rabbit	SC; sc-15378	1:150
GATA-6	rabbit	Cell Signaling; 5851	1:200
GM130	mouse	BD transduction; 610823	1:300
GR	rabbit	SC; sc-8992	1:250
GS28	mouse	BD transduction; 611184	1:200
Hsc70	mouse	StressMarq; SMC-151	1:200
LAMP1	mouse	DHSB; H4A3	1:400
LC3B	rabbit	abcam; ab48394	1:200
Nucleoporin p62	mouse	BD transduction; 610497	1:200
Sec61ß	rabbit	custom-made; Berhard Dobberstein, Heidelberg	1:300
SGTA	chicken	custom-made	1:400
Stx5	rabbit	SySy; 110053	1:200
**secondary antibody**	**host species**	**company**	**dilution**
anti-Rabbit IgG (Alexa Fluor 488)	goat	Invitrogen, A11034	1:1000
anti-Mouse IgG (Alexa Fluor 647)	goat	Invitrogen, A21235	1:1000
anti-Chicken IgY (Alexa Fluor 647)	goat	Invitrogen, A21449	1:1000

Imaging with a LSM 510-META confocal microscope (Zeiss)

HeLa P4 cells were analysed using an Axiovert 200M fluorescence microscope with a 63× Plan-Neofluar 1.3 NA water-corrected objective and appropriate filter settings. Images were taken using a LSM 510-META confocal laser scanning microscope (Zeiss). For confocal imaging a UV laser (405 nm), a tunable Argon laser (488 nm) and HeNe laser lines (633 nm) were used for excitation.

Ilaria Testa

Immunohistochemistry of U2OS cells and primary hippocampal neurons (Figure 14)

U2OS cells were cultured in DMEM, supplemented with 1% penicillin/streptomycin and 10% fetal bovine serum, and seeded on 18mm coverslips.

Primary hippocampal cultures were prepared from E18 Sprague Dawley rat embryos and cultured on poly-ornithine coated 18mm coverslips, under normal conditions. The animal experiments were approved by the Institutional animal care and use committee of the Karolinska Institutet.

For the imaging of endoplasmic reticulum, U2OS cells were transfected with an EGFP-KDEL plasmid using Lipofectamine LTX with Plus reagent (ThermoFisher Scientific), following the instruction manual from the company. 24h after transfection the cells were fixed with either PFA 4% or glyoxal 3% solution (pH5), following the protocol described above (30min on ice + 30min at RT). Afterwards, the cells were quenched with 100 mM NH_4Cl for 20 min; permeabilization and blocking was done for 15

min in 2.5 % BSA and 0.1 % Triton X-100 in PBS. The cells were then incubated for 60 min with the primary antibodies: Rabbit polyclonal against GFP (1:100, Abcam, ab6556) or Tom20 Antibody (1:100, Santa Cruz Biotechnology, sc-11415). After washing in blocking solution for 15 min, the cells were incubated with the secondary antibody for 60 min: Star Red (1:200, Abberior). Subsequent washing in phosphate buffer saline was followed by embedding in Mowiol.

Primary hippocampal neurons after 2 days of culture were fixed with either PFA 4% or glyoxal 3% solution (pH5), following the protocol described above (60 min at RT), and were subsequently quenched for 30 min in 100 mM NH_4Cl. Permeabilization was achieved by incubating the neurons for 15 min in blocking solution, containing 2.5 % BSA and 0.1 % Triton X-100 in PBS. The samples were then incubated with the Sodium / Potassium ATPase alpha-3 Antibody (ThermoFisherScientific, MA3-915) for 60 min at RT. After washing 15 min in blocking solution, Star Red secondary antibody (Abberior) was applied for 60 min, at room temperature. Subsequent washing in high salt PBS (500 mM NaCl) and PBS was followed by embedding in Mowiol.

Imaging with a custom STED/confocal

The confocal images of KDEL, Tom20 and Sodium / Potassium ATPase alpha-3 were recorded with a custom built STED/confocal microscope equipped with a 100X/1.4 oil objective, using a 640 nm laser line to excite STAR RED and a 775 nm laser to deplete the signal.

Bolek Zapiec

Mice

Mouse experiments were performed in accordance with the German Animal Welfare Act, European Communities Council Directive 2010/63/EU, and the institutional ethical and animal welfare guidelines of the Max Planck Research Unit for Neurogenetics. Approval came from the Regierungspräsidium Darmstadt and the Veterinäramt of the City of Frankfurt. C57BL6/J female littermates at 3 weeks were anesthetized with an intraperitoneal injection of ketamine/xylazin sodium chloride (NaCl) solution (210 mg/kg ketamine, 10 mg/kg xylazin). Mice were perfused with 12 ml 0.9% NaCl solution, and then with either 24 ml 4% paraformaldehyde (PFA) in Phosphate Buffer Saline (PBS) or 24 ml 3% pH 4 glyoxal fixative solution, both freshly made just prior to use. The olfactory mucosa and olfactory bulbs were dissected and post-fixed overnight at 4˚C before washing in PBS 3 times for 10 minutes, decalcifying in 450 mM EDTA in PBS at 4˚C overnight, incubating in 15% sucrose in PBS at 4˚C for 4 h, in 30% sucrose in PBS at 4˚C overnight, and embedding in OCT medium (TissueTek).

Immunohistochemistry (Figure 15)

Frozen blocks of mouse olfactory tissue were sectioned at 12 µm thickness and collected on SuperFrost Ultra Plus slides (ThermoFisher). The slides were washed 3 times for 5 min with PBS at RT, then they were blocked in 10% NDS in 0.2% Triton X-100 PBS (PBST) at RT for 2 h. The blocking solution was tipped off the slides, and the sections were incubated in a primary antibody solution containing 3% NDS in PBST overnight at 4˚C. Slides were then washed 3 times in PBST for 10 min at RT and incubated in a secondary antibody solution containing 3% NDS in PBS. The slides were subsequently washed 3 times in PBS for 10 min each and stained with DAPI at 1:10,000 in PBS for 10 minutes before being washed 3 times for 5 minutes in PBS. The primary antibodies (1:500) were goat anti-OMP (Wako, 544-10001-WAKO), mouse monoclonal anti-β3-tubulin (Sigma T8660), rabbit monoclonal anti-neuropilin-1 (Abcam, AB81321), goat anti-neuropilin-2 (R&D Systems, AF567), guinea pig anti-VGLUT2 (Synaptic Systems, 135 404), and rabbit anti-VGLUT2 (Synaptic Systems, 135 403). The secondary antibodies (1:500) were donkey anti-goat Alexa488 (ThermoFisher, A11055), donkey anti-rabbit Alexa546 (ThermoFisher, A10040), and donkey anti-mouse Cy5 (Jackson ImmunoResearch, 715-175-150). The slides were then imaged on a Zeiss LSM 710 confocal microscope.

References

Bethani, I., Lang, T., Geumann, U., Sieber, J. J., Jahn, R., & Rizzoli, S. O. (2007). The specificity of SNARE pairing in biological membranes is mediated by both proof-reading and spatial segregation. *The EMBO journal*, *26*(17), 3981–92. doi:10.1038/sj.emboj.7601820

Charneau P, Mirambeau G, Roux P, Paulous S, Buc HCF (1994) HIV-1 reverse transcription. A termination step at the center of the genome. J Mol Biol 241: 651 – 662

Lang, T., & Rizzoli, S. O. (2010a). Membrane protein clusters at nanoscale resolution: more than pretty pictures. *Physiology (Bethesda, Md.)*, *25*(2), 116–24. doi:10.1152/physiol.00044.2009

Löschberger, A., van de Linde, S., Dabauvalle, M.-C., Rieger, B., Heilemann, M., Krohne, G., & Sauer, M. (2012). Super-resolution imaging visualizes the eightfold symmetry of gp210 proteins around the nuclear pore complex and resolves the central channel with nanometer resolution. *Journal of cell science*, *125*(Pt 3), 570–5. doi:10.1242/jcs.098822

Opazo, F., Levy, M., Byrom, M., Schäfer, C., Geisler, C., Groemer, T. W., … Rizzoli, S. O. (2012). Aptamers as potential tools for super-resolution microscopy. *Nature methods*, *9*(10), 938–9. doi:10.1038/nmeth.2179

Opazo, F., Punge, A., Bückers, J., Hoopmann, P., Kastrup, L., Hell, S. W., & Rizzoli, S. O. (2010). Limited intermixing of synaptic vesicle components upon vesicle recycling. *Traffic (Copenhagen, Denmark)*, *11*(6), 800–12. doi:10.1111/j.1600-0854.2010.01058.x

Revelo, N. H., Kamin, D., Truckenbrodt, S., Wong, A. B., Reuter-Jessen, K., Reisinger, E., … Rizzoli, S. O. (2014). A new probe for super-resolution imaging of membranes elucidates trafficking pathways. *The Journal of cell biology*, *205*(4), 591–606. doi:10.1083/jcb.201402066

Wagner E, Lauterbach MA, Kohl T, Westphal V, Williams GSB, Steinbrecher JH, Streich J-H, Korff B, Tuan H-TM, Hagen B, Luther S, Hasenfuss G, Parlitz U, Jafri MS, Hell SW, Lederer WJ, Lehnart SE (2012) Stimulated emission depletion live-cell super-resolution imaging shows proliferative remodeling of T-tubule membrane structures after myocardial infarction. Circ Res 111: 402 – 414

Wagner E, Brandenburg S, Kohl T, Lehnart SE (2014) Analysis of tubular membrane networks in cardiac myocytes from atria and ventricles. J Vis Exp 92: e51823

Wilhelm, B. G., Mandad, S., Truckenbrodt, S., Kröhnert, K., Schäfer, C., Rammner, B., … Rizzoli, S. O. (2014). Composition of isolated synaptic boutons reveals the amounts of vesicle trafficking proteins. *Science (New York, N.Y.)*, *344*(6187), 1023–8. doi:10.1126/science.1252884
