# Supplementary material for: Glyoxal as an alternative fixative to formaldehyde in immunostaining and super‐resolution microscopy
Source: EMBO J. 2017 Nov 16;37(1):139–59. doi: 10.15252/embj.201695709 (PMC5753035; doi:10.15252/embj.201695709)
Supplement: Supplementary file 2 — Source Data for Appendix [file EMBJ-37-139-s002.zip › SourceDataForFigureS12.pdf]

Source data for Appendix Figure 12

α/β SNAP

PFA

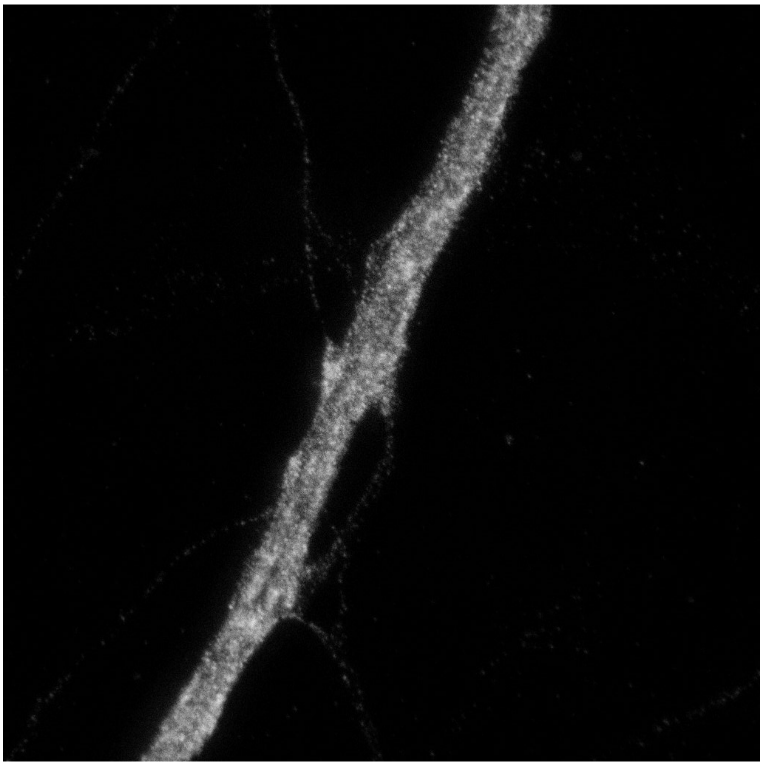

Glyoxal

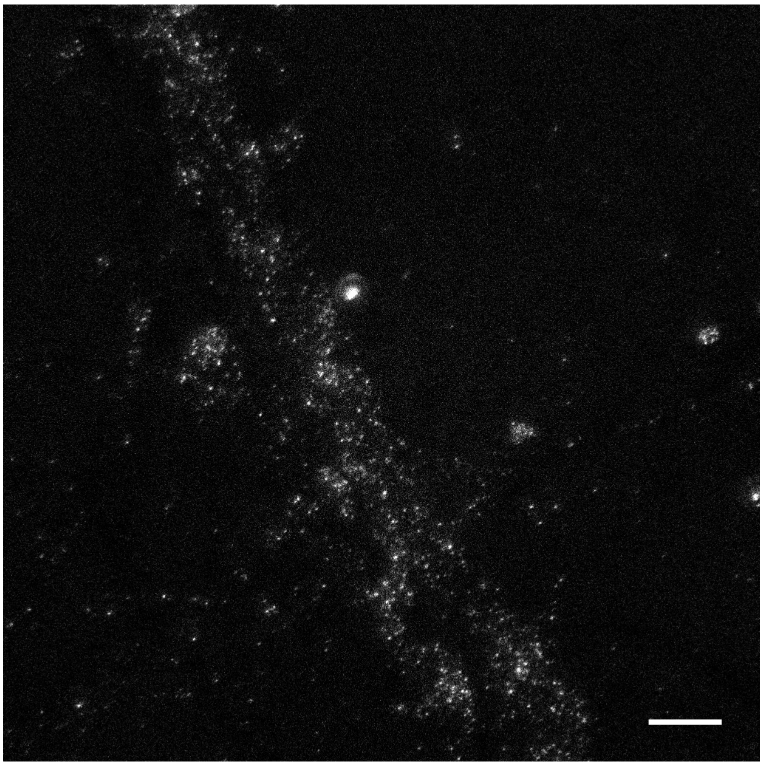

α-tubulin

PFA

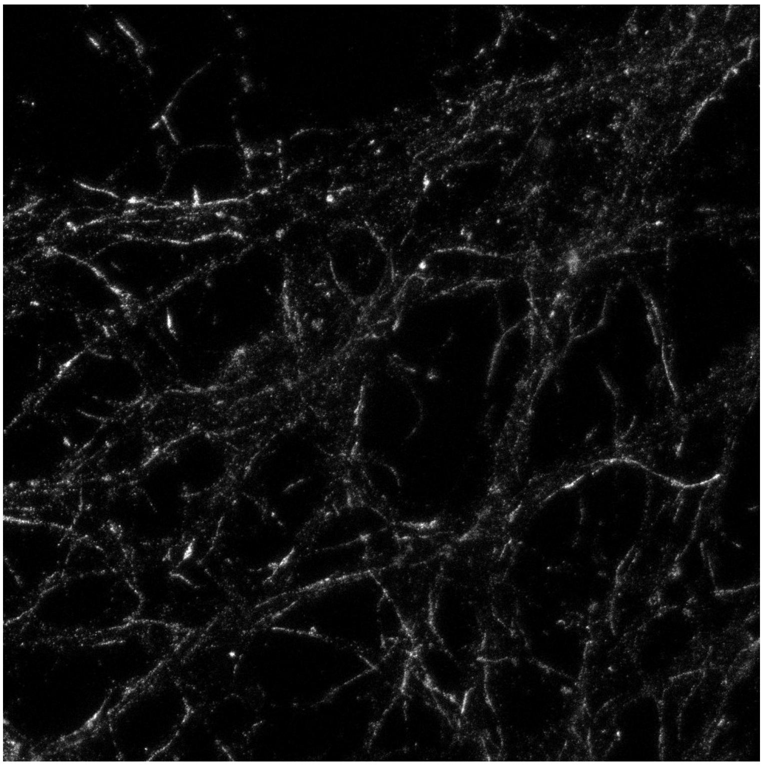

Glyoxal

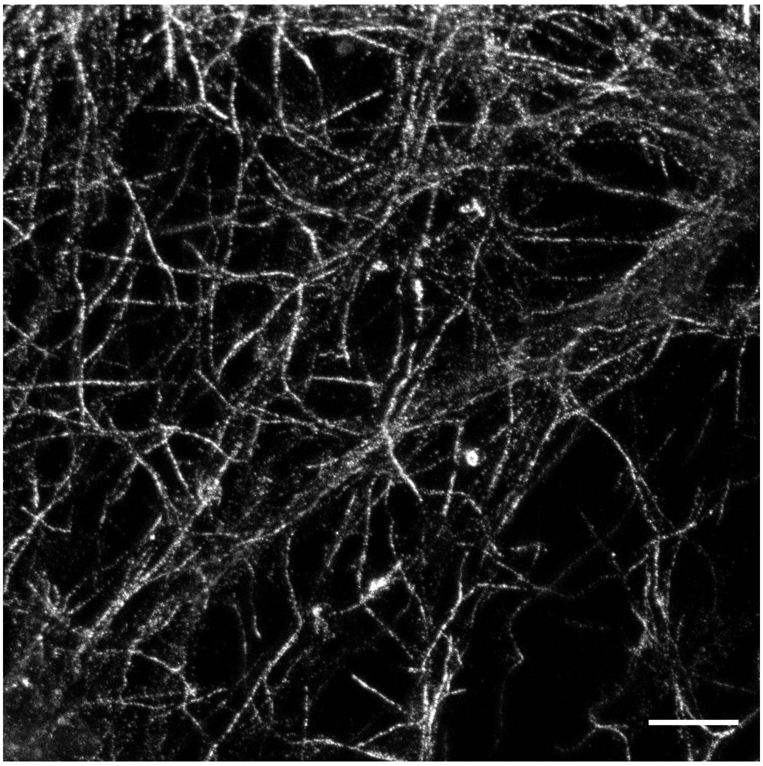

β-actin

PFA

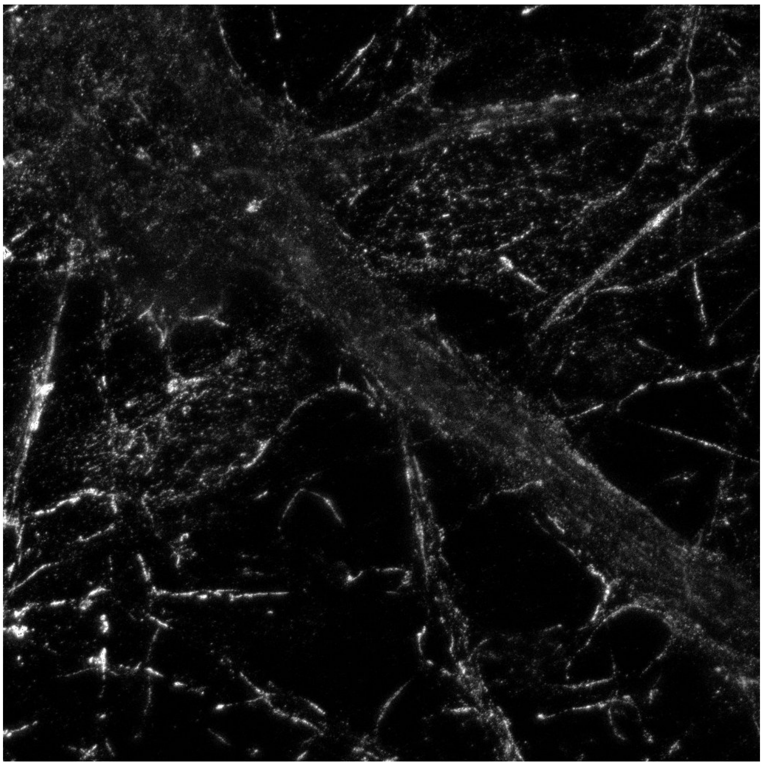

Glyoxal

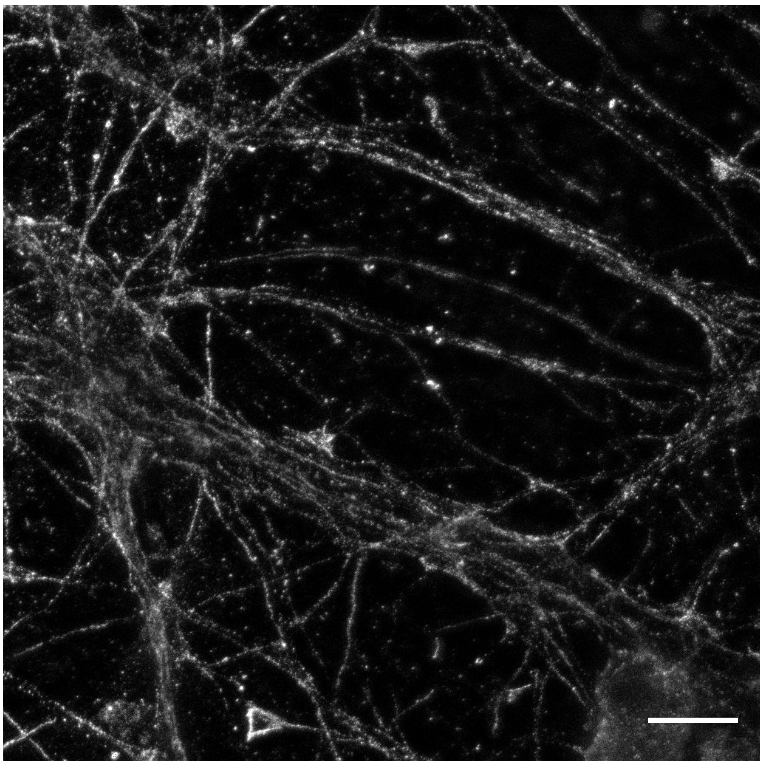

Bassoon

PFA

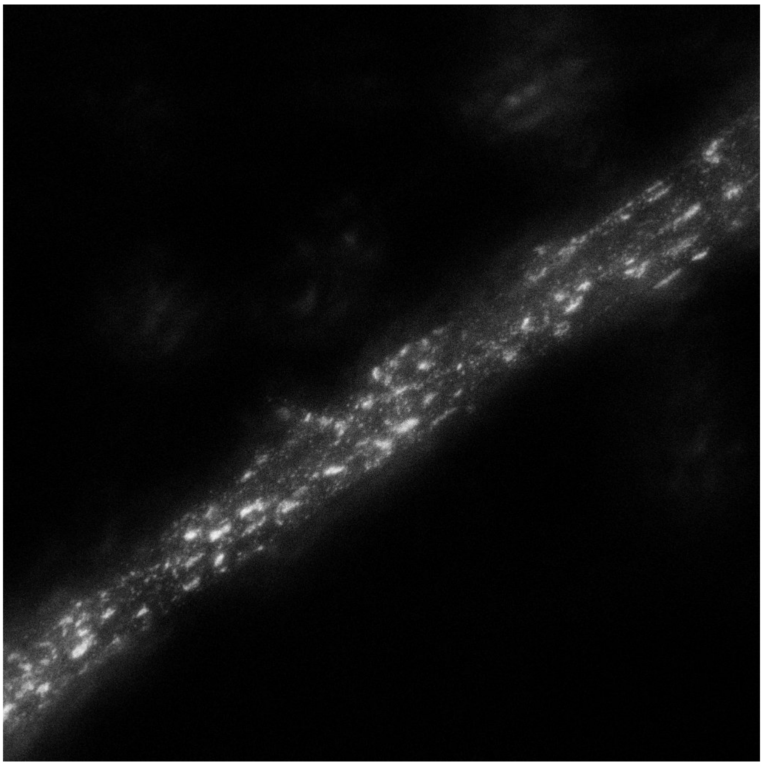

Glyoxal

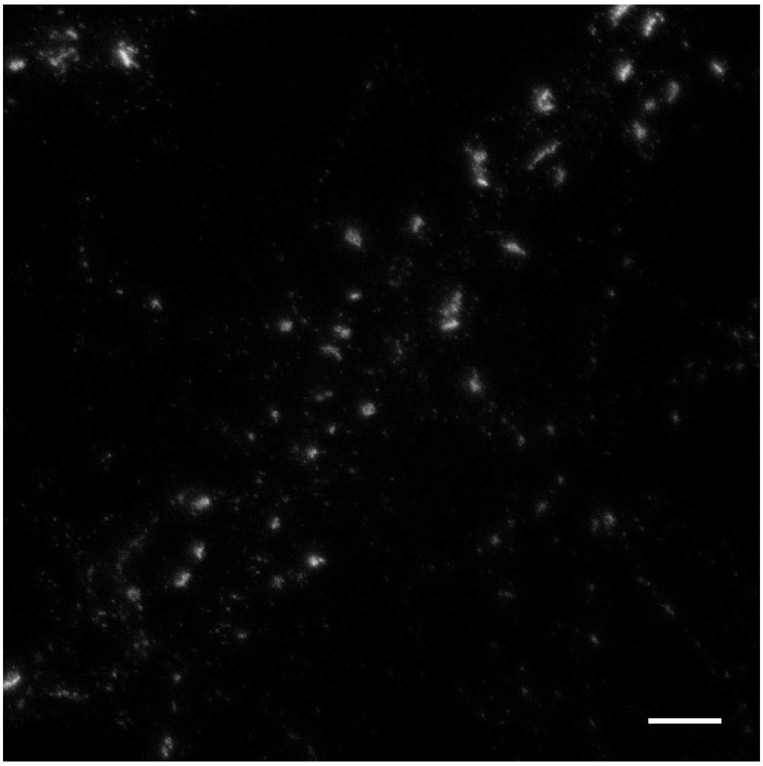

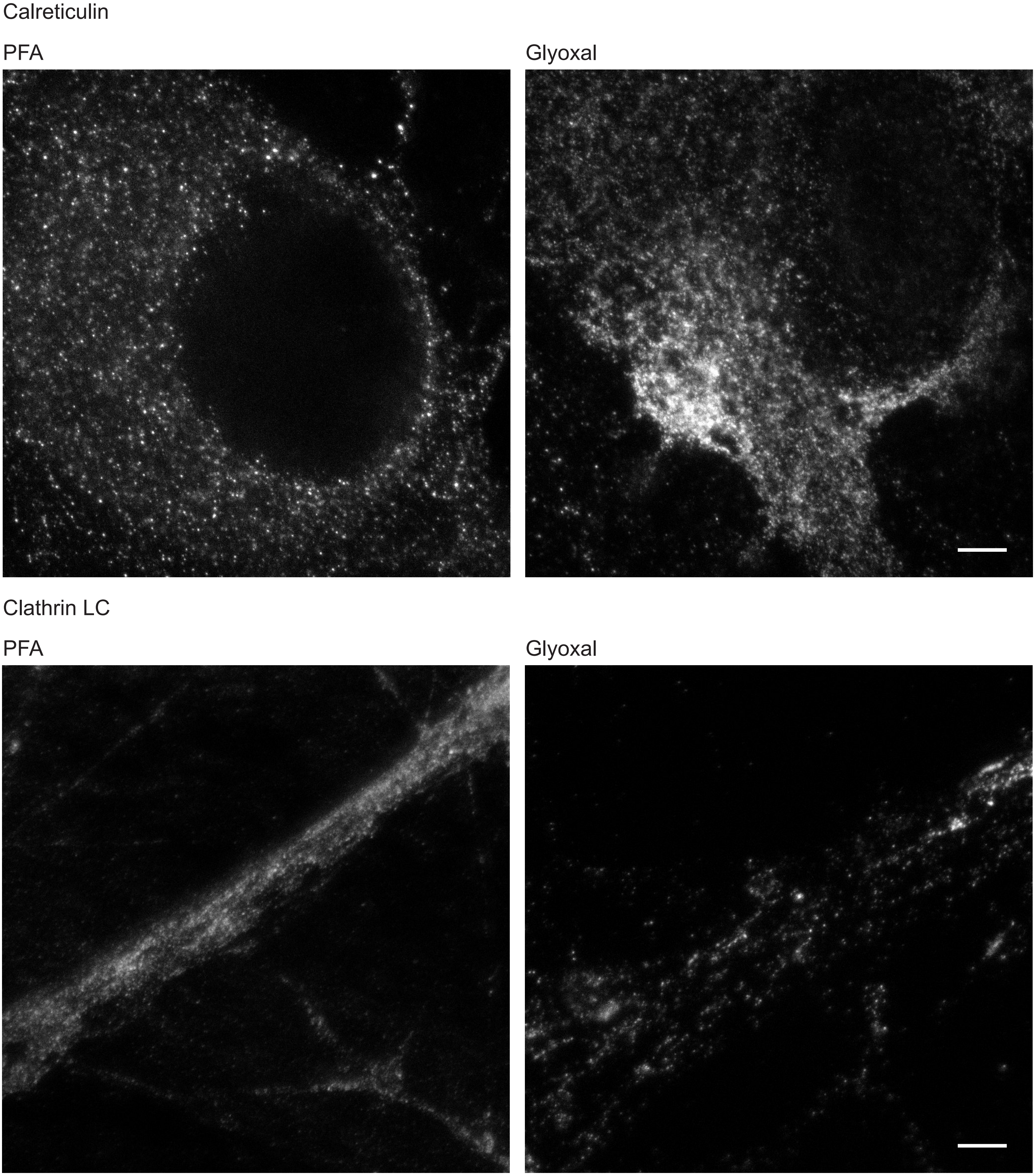

Calreticulin
PFA
Glyoxal
Clathrin LC
PFA
Glyoxal

HSC70

PFA

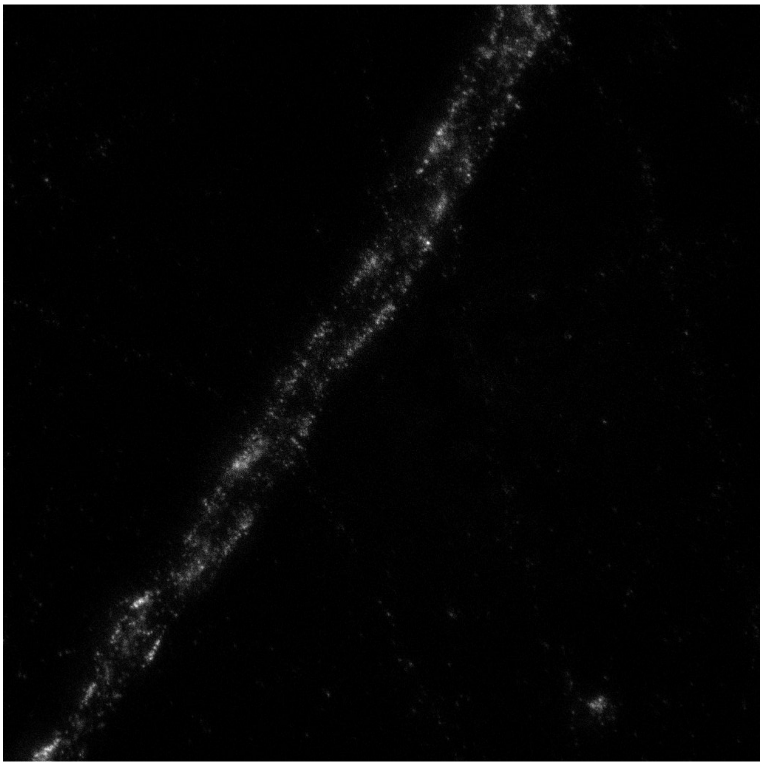

Glyoxal

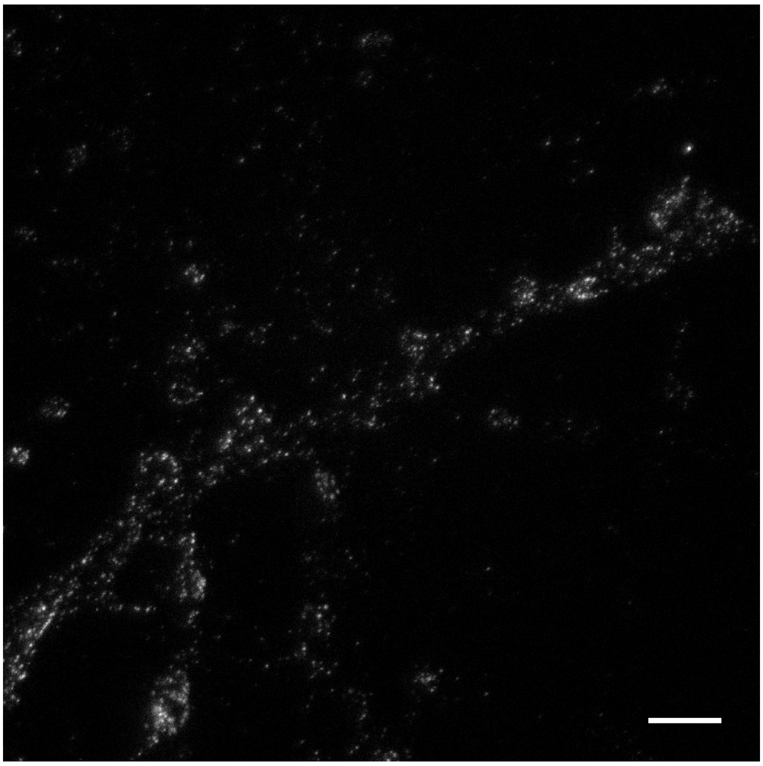

Neurofilament L

PFA

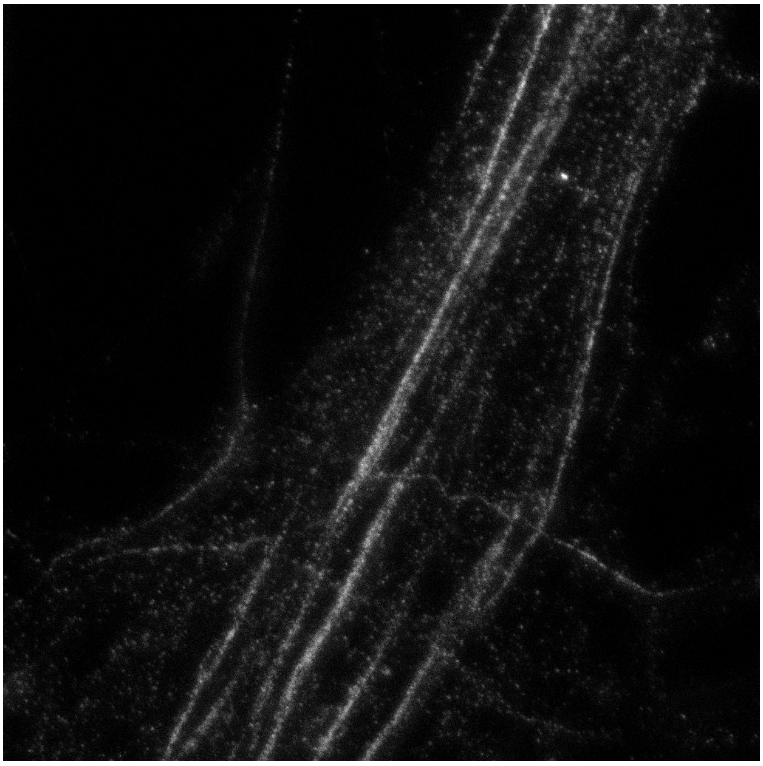

Glyoxal

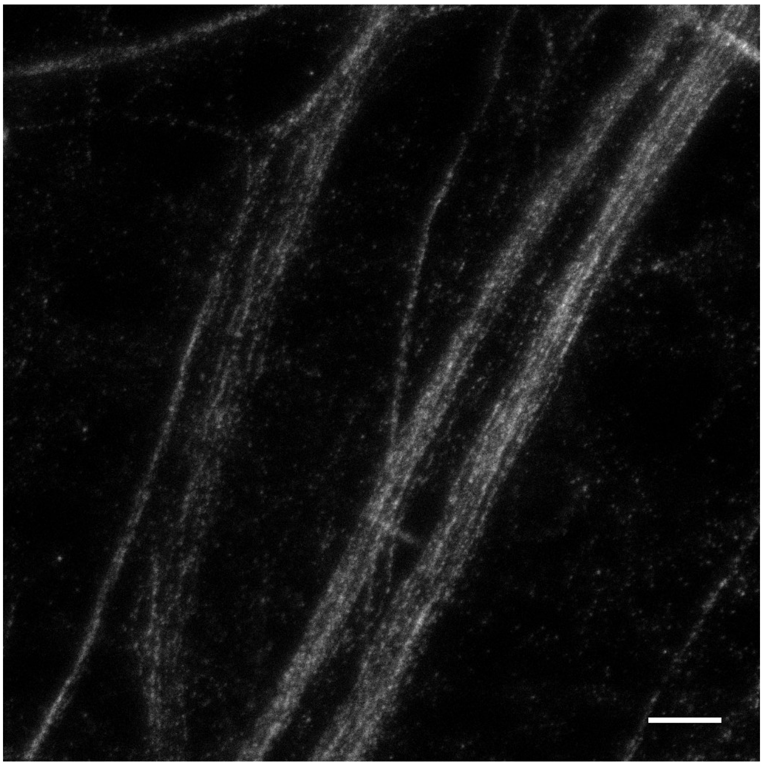

NSF

PFA

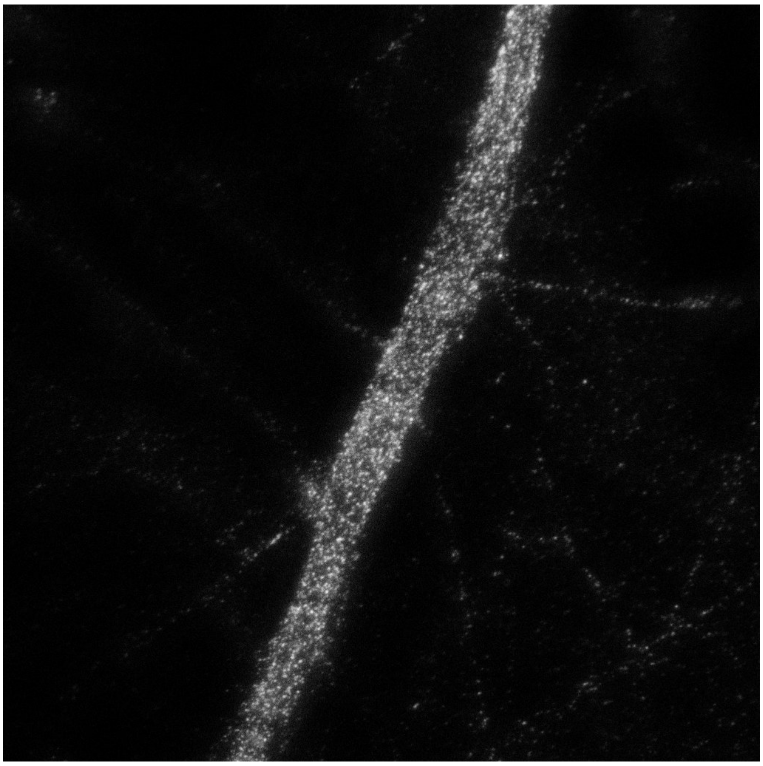

Glyoxal

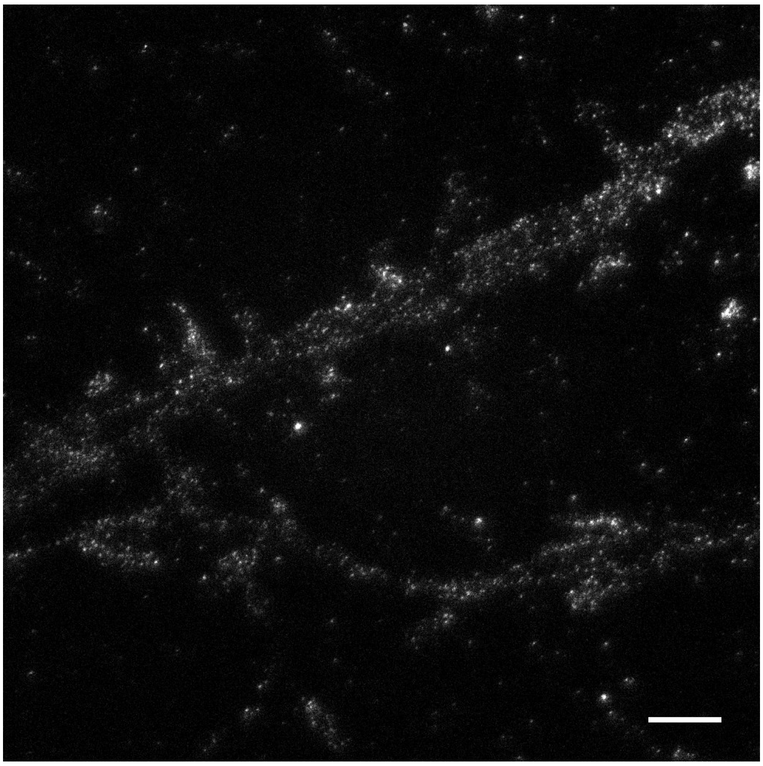

PSD95

PFA

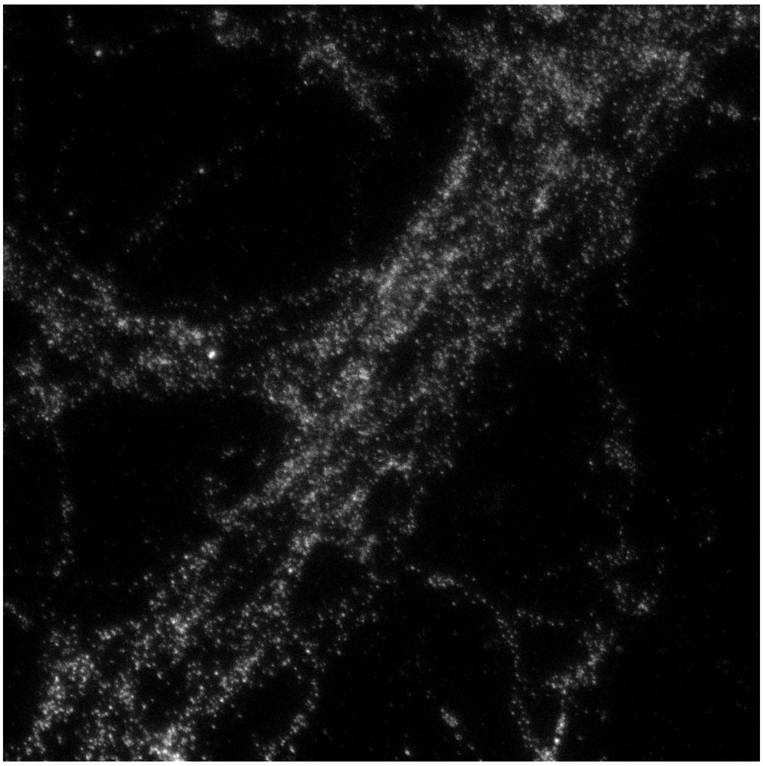

Glyoxal

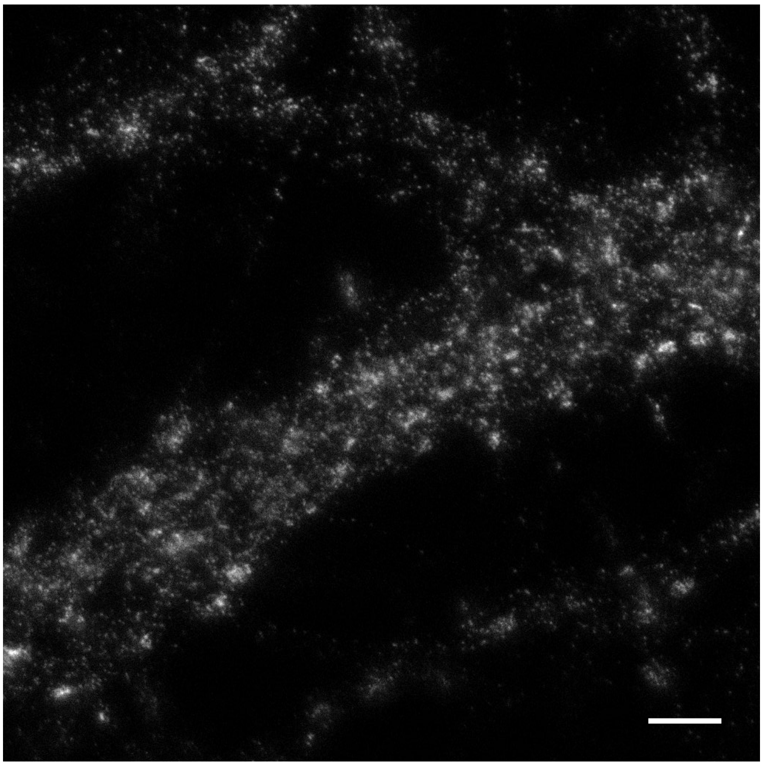

Rab5

PFA

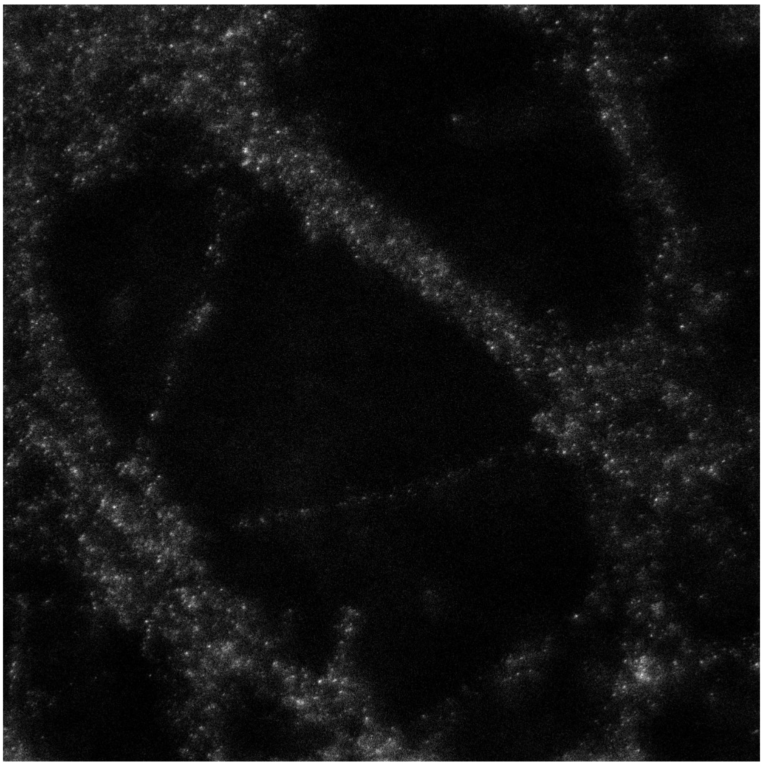

Glyoxal

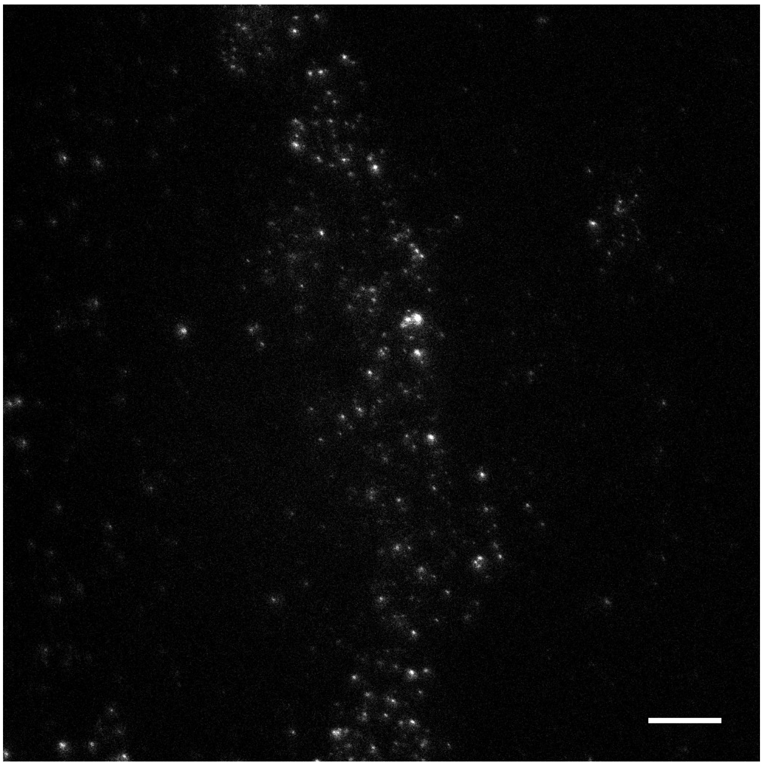

Rab7

PFA

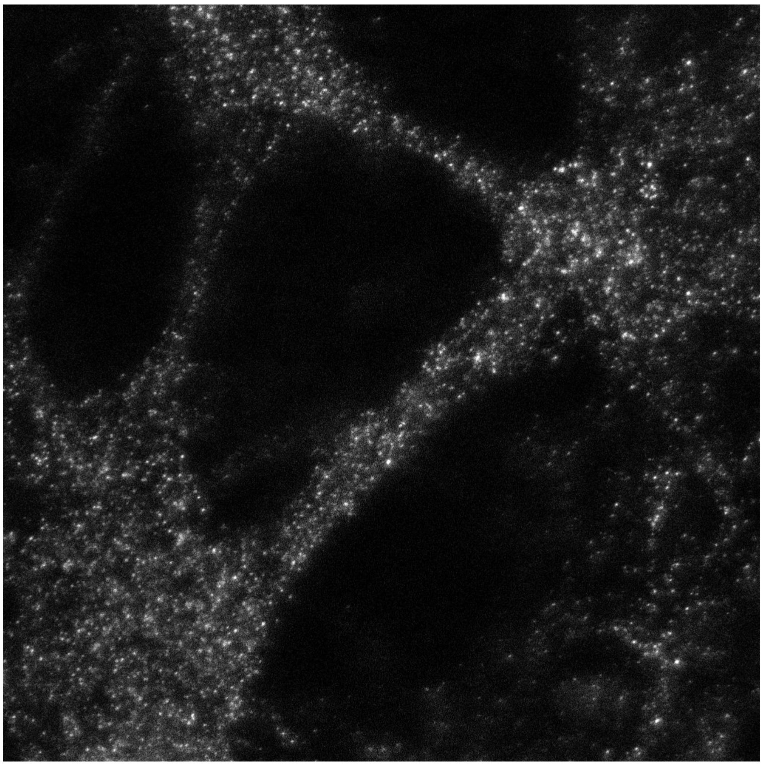

Glyoxal

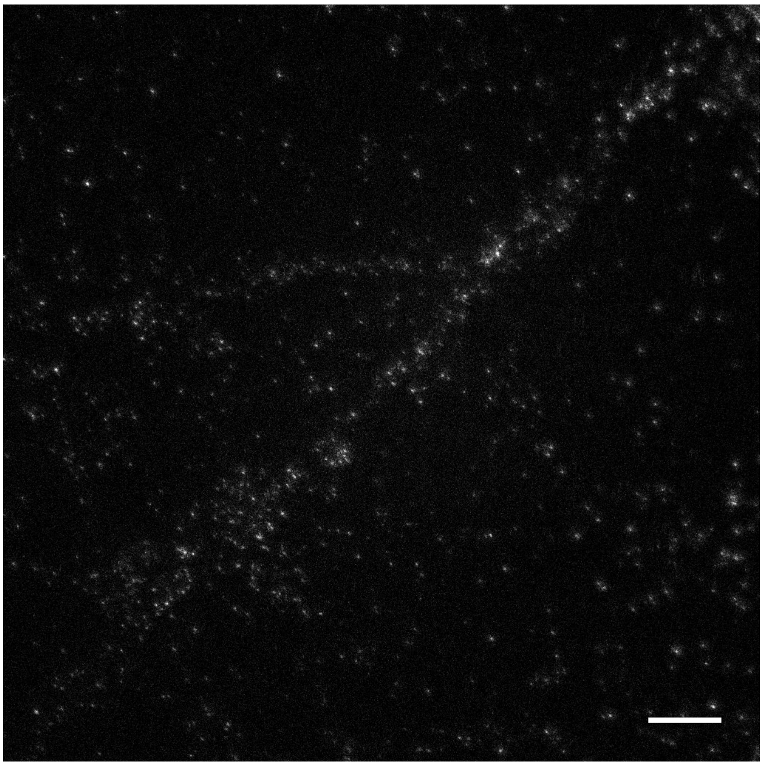

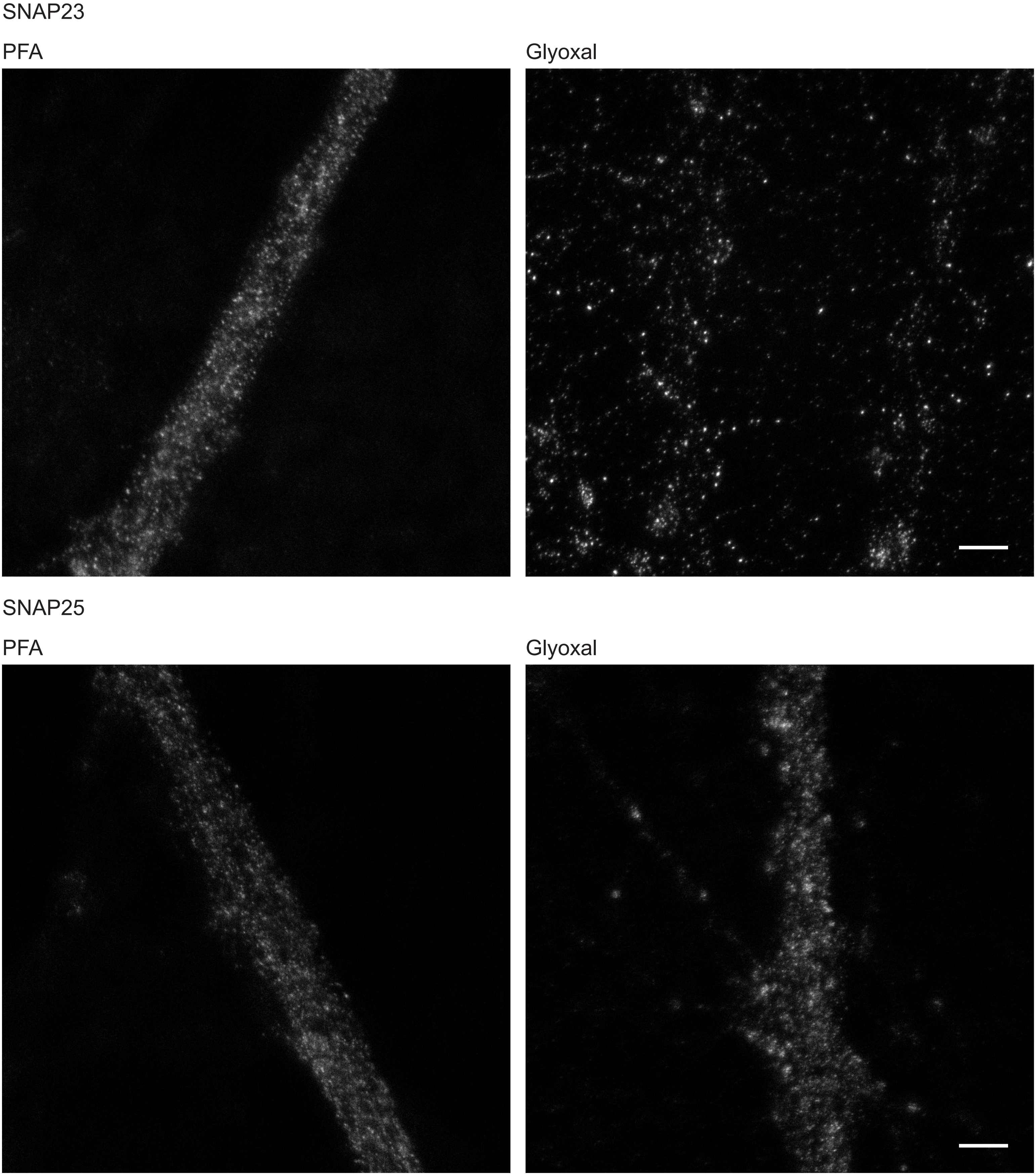
SNAP23
PFA
Glyoxal
SNAP25
PFA
Glyoxal

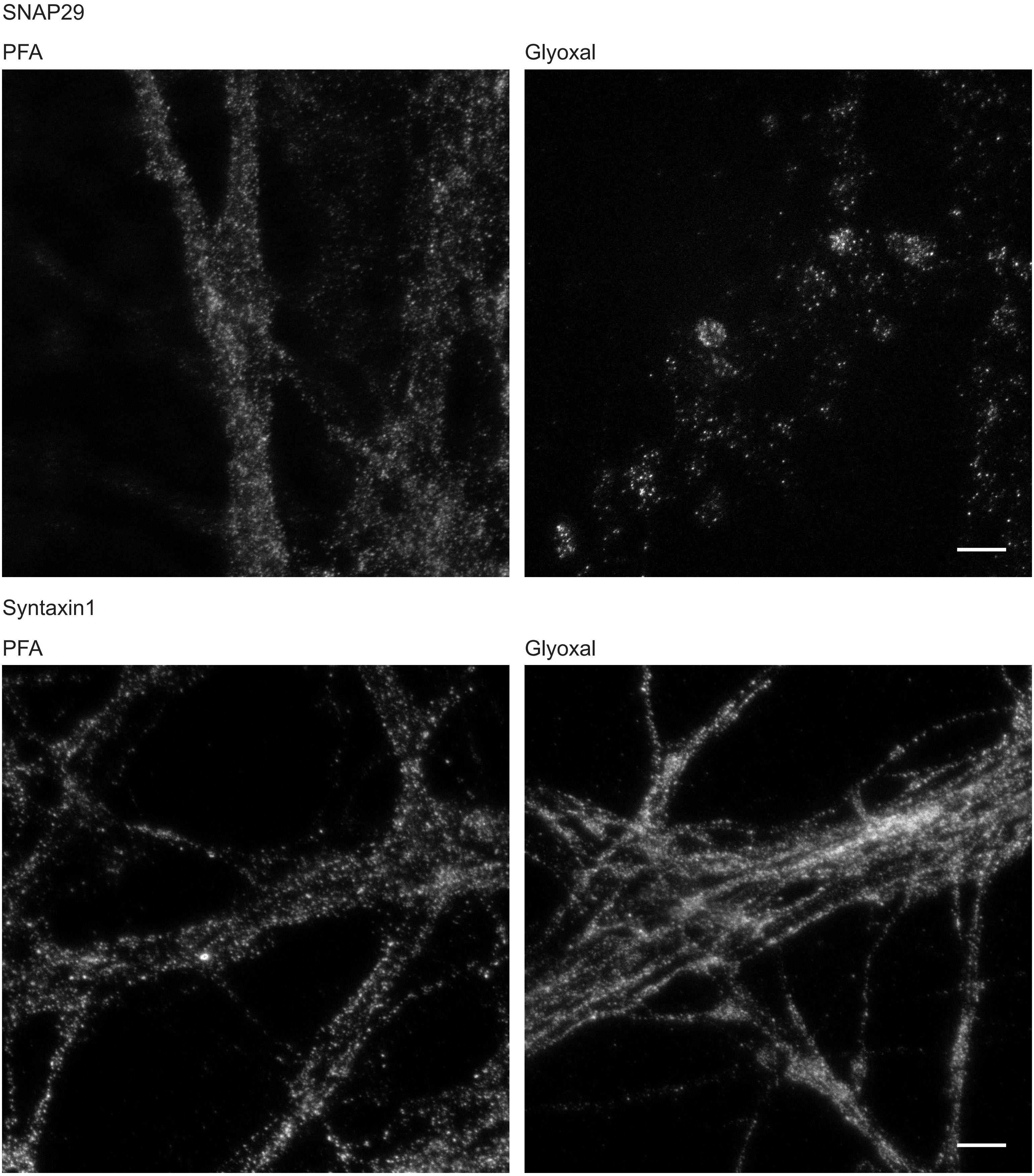
SNAP29
PFA
Glyoxal
Syntaxin1
PFA
Glyoxal

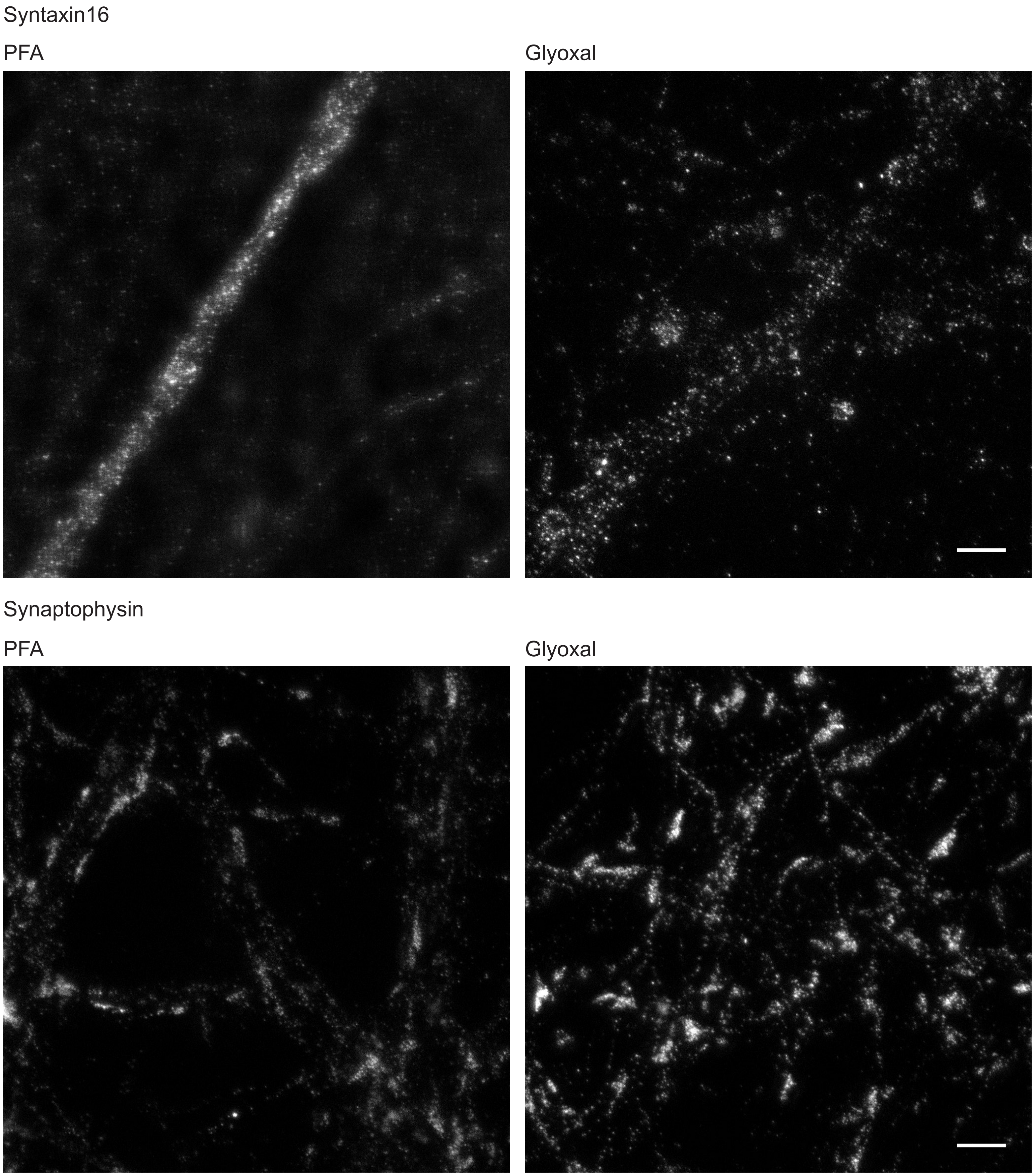
Syntaxin16
PFA
Glyoxal
Synaptophysin
PFA
Glyoxal

Synaptotagmin7

PFA

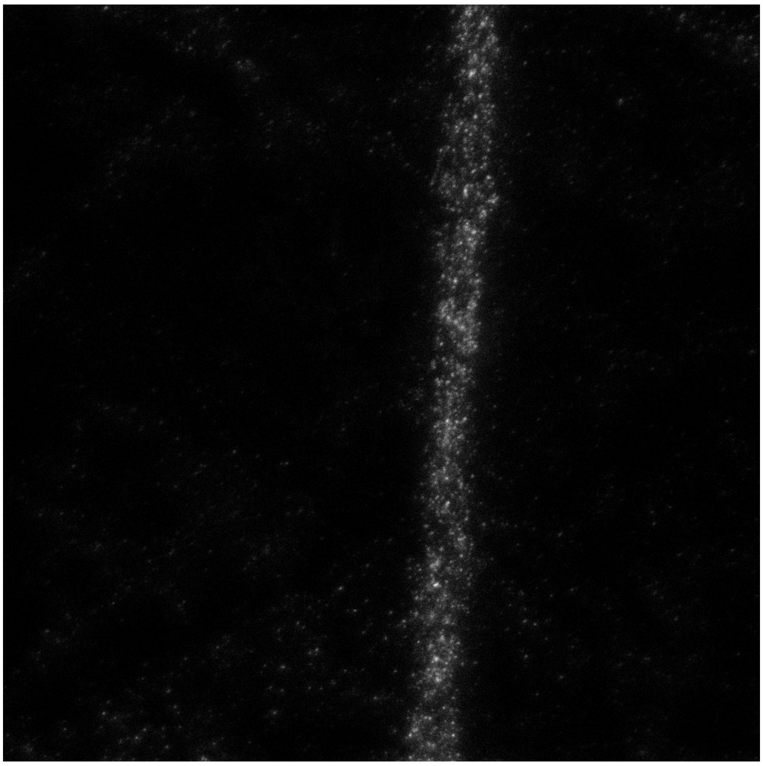

Glyoxal

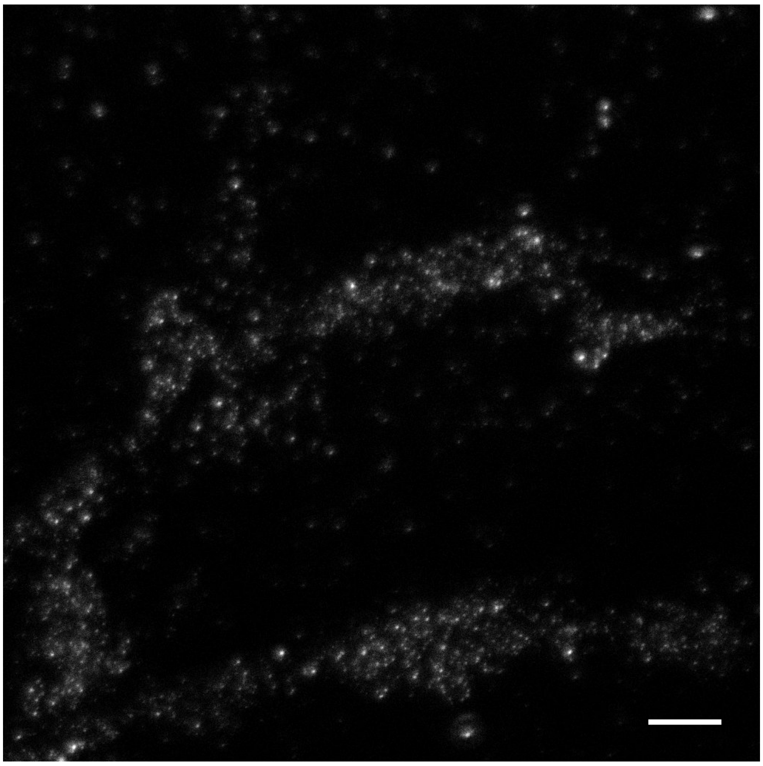

VAMP2

PFA

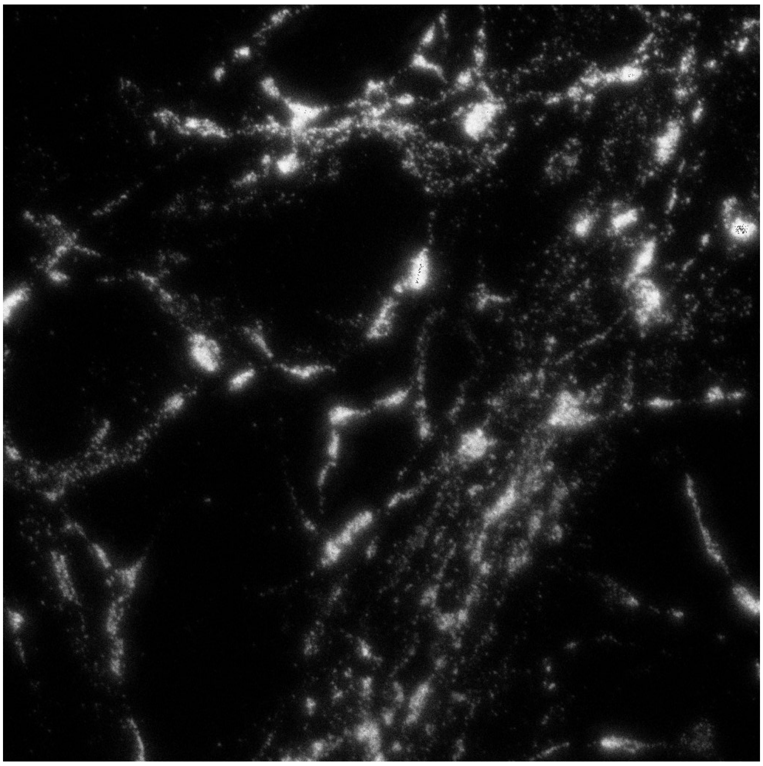

Glyoxal

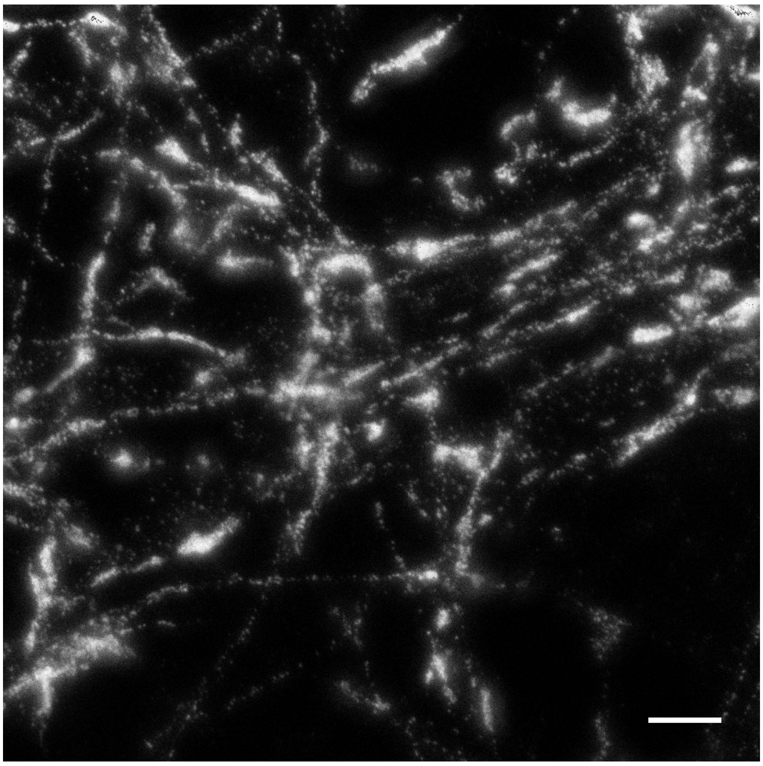

Full size STED images of immunolabeled neuronal proteins from Appendix Figure S12
Images were adjusted in contrast and brightness using ImageJ. No further image processing was applied. Scale bar = 6 µm for β-actin and α-tubulin, and 2 µm for the rest.
